# Hypoxia Promotes Atrial Tachyarrhythmias via Opening of ATP-Sensitive Potassium Channels

**DOI:** 10.1161/CIRCEP.123.011870

**Published:** 2023-08-30

**Authors:** Mark J. Specterman, Qadeer Aziz, Yiwen Li, Naomi A. Anderson, Leona Ojake, Keat-Eng Ng, Alison M. Thomas, Malcolm C. Finlay, Richard J. Schilling, Pier D. Lambiase, Andrew Tinker

**Affiliations:** William Harvey Research Institute, Barts and the London School of Medicine and Dentistry, Queen Mary University of London, United Kingdom (M.J.S., Q.A., Y.L., N.A.A., L.O., K.-E.N., A.M.T., M.C.F., R.J.S., A.T.).; Institute of Cardiovascular Science, University College London, United Kingdom (P.D.L.).

**Keywords:** atrial fibrillation, cardiac arrhythmia, hypoxia, ischemia, K_ATP_ channels

## Abstract

**BACKGROUND:**

Hypoxia-ischemia predisposes to atrial arrhythmia. Atrial ATP-sensitive potassium channel (K_ATP_) modulation during hypoxia has not been explored. We investigated the effects of hypoxia on atrial electrophysiology in mice with global deletion of K_ATP_ pore-forming subunits.

**METHODS:**

Whole heart K_ATP_ RNA expression was probed. Whole-cell K_ATP_ current and action potentials were recorded in isolated wild-type (WT), Kir6.1 global knockout (6.1-gKO), and Kir6.2 global knockout (6.2-gKO) murine atrial myocytes. Langendorff-perfused hearts were assessed for atrial effective refractory period (ERP), conduction velocity, wavefront path length (WFPL), and arrhymogenicity under normoxia/hypoxia using a microelectrode array and programmed electrical stimulation. Heart histology was assessed.

**RESULTS:**

Expression patterns were essentially identical for all K_ATP_ subunit RNA across human heart, whereas in mouse, Kir6.1 and SUR2 (sulphonylurea receptor subunit) were higher in ventricle than atrium, and Kir6.2 and SUR1 were higher in atrium. Compared with WT, 6.2-gKO atrial myocytes had reduced tolbutamide-sensitive current and action potentials were more depolarized with slower upstroke and reduced peak amplitude. Action potential duration was prolonged in 6.1-gKO atrial myocytes, absent of changes in other ion channel gene expression or atrial myocyte hypertrophy. In Langendorff-perfused hearts, baseline atrial ERP was prolonged and conduction velocity reduced in both K_ATP_ knockout mice compared with WT, without histological fibrosis. Compared with baseline, hypoxia led to conduction velocity slowing, stable ERP, and WFPL shortening in WT and 6.1-gKO hearts, whereas WFPL was stable in 6.2-gKO hearts due to ERP prolongation with conduction velocity slowing. Tolbutamide reversed hypoxia-induced WFPL shortening in WT and 6.1-gKO hearts through ERP prolongation. Atrial tachyarrhythmias inducible with programmed electrical stimulation during hypoxia in WT and 6.1-gKO mice correlated with WFPL shortening. Spontaneous arrhythmia was not seen.

**CONCLUSIONS:**

K_ATP_ block/absence leads to cellular and tissue level atrial electrophysiological modification. Kir6.2 global knockout prevents hypoxia-induced atrial WFPL shortening and atrial arrhythmogenicity to programmed electrical stimulation. This mechanism could be explored translationally to treat ischemically driven atrial arrhythmia.

**WHAT IS KNOWN?**

- There is an association between atrial ischemia and the propensity to atrial arrhythmias, although the mechanism is unclear.
- Cardiac ATP-sensitive potassium channel (K_ATP_) activation can lead to an increased fibrillatory potential and the pore-forming subunit of cardiac sarcolemmal K_ATP_ is thought to be Kir6.2.

**WHAT THE STUDY ADDS**

- Kir6.2 is the major pore-forming subunit of K_ATP_ currents in murine atria, although Kir6.1 also contributes to atrial electrophysiology and Kir6.1 is expressed in human atria.
- K_ATP_ activation during hypoxia-ischemia enables maintenance of the atrial effective refractory period, wavefront path length shortening through conduction slowing, and increased atrial arrhythmia inducibility to programmed electrical stimulation.
- This provides a mechanism of hypoxia-ischemia-induced atrial arrhythmia and K_ATP_ is worthy of investigation as a druggable target in this regard.

Atrial fibrillation (AF) is the commonest cardiac arrhythmia, and frequently complicates management when clinically observed in conditions of myocardial stress such as during ischemia, critical illness, or cardiac surgery.^1^ Atrial ischemia can lead to episodes of AF and their perpetuation, and this is likely to be via direct electrophysiological effects.^2^

One of the key ion channels sensitive to cellular metabolism are ATP-sensitive potassium channels (K_ATP_) and they are abundantly expressed in the heart. K_ATP_ channels open in response to a drop in the intracellular ATP:ADP ratio during ischemia.^3^ A functional channel consists of a hetero-octamer of subunits: 4 pore-forming subunits (Kir6.1 or Kir6.2) and 4 regulatory SUR (sulphonylurea receptor) subunits (SUR1, SUR2A, or SUR2B).^4^ An array of openers and inhibitors act selectively at the SUR subunit.^5^

In rodents, a differential K_ATP_ sarcolemmal subunit composition has been shown between the atria (Kir6.2/SUR1) and ventricles (Kir6.2/SUR2A), although this has not been investigated exhaustively, particularly with regards to the pore-forming subunits.^6^ Thus far, the same differential subunit composition has not been demonstrated in human heart.^7^ However, this has not been fully dissected and Kir6.1 may represent a more important cardiac subunit in humans.

The opening of cardiac sarcolemmal K_ATP_ channels during metabolic stress leads to action potential (AP) shortening and reduced calcium influx reducing the energy requirements of the myocyte.^3^ In this manner, heterogeneity of AP shortening increases the propensity to reentrant arrhythmias and a profibrillatory state, although the majority of data pertain to the ventricle.^8^ In a rat model, β-adrenergic-induced metabolic stress led to reduced intracellular ATP concentration and inducible atrial tachyarrhythmia reversed with K_ATP_ inhibiting drugs.^9^ In a murine model of salt-induced hypertension, parallel upregulation of atrial K_ATP_ was seen with shortened effective refractory period (ERP) and increased atrial arrhythmia inducibility.^10^ K_ATP_ activating drugs led to increased atrial arrhythmia inducibility, then reversed with K_ATP_ inhibition in human hearts obtained at transplantation.^7^

An alternative paradigm is that membrane hyperpolarization and stabilization may also be antiarrhythmic with reduced propensity to automatic and triggered arrhythmia.^11^ Simultaneous optical AP and calcium transient measurement in the intact heart has supported the importance of Kir6.2 and SUR1 in the atria in a knockout murine model where a failure of AP and calcium transient shortening with metabolic poisoning led to focal ventricular activity indicative of afterdepolaristions.^12,13^ The consequent mechanisms of hypoxia/ischemia-induced K_ATP_ channel opening on atrial arrhythmogenicity have not been fully investigated.

Thus, we explored the electrophysiological sequelae of K_ATP_ modulation and the contribution of each pore subunit by performing a systematic comparison of global knockout mice with deletion of each of the K_ATP_ pore-forming subunits.

## METHODS

The data that support the findings of this study are available from the corresponding author on reasonable request.

### Molecular Biology and Cell Culture

cDNAs encoding the Kir6x pore-forming and SURx sulphonylurea receptor subunits and human embryonic kidney (HEK) 293 cells stably transfected with Kir6.1/SUR1, Kir6.2/SUR1, Kir6.1/SUR2A, Kir6.2/SUR2A, Kir6.1/SUR2B, and Kir6.2/SUR2B were used as described previously.^14^

### Animal Husbandry

All experiments were conducted in accordance with the Guide for the Care and Use of Laboratory Animals published by the British Home Office and the US National Institutes of Health (Publication 85-23, revised 1996). Animal work was approved by the Queen Mary University of London Animal Welfare and Ethical Review Body and covered by British Home Office Project Licences PPL/7665 and PE9055EAD.

### Generation of Kir6x Global Knockout Mice

Detailed methods are given in the Supplemental Material.

### Quantitative Reverse Transcription Polymerase Chain Reaction

Detailed methods are given in the Supplemental Material.

### Histology and Immunohistochemistry

Detailed methods are given in the Supplemental Material.

### Isolation and Patch Clamp Electrophysiology of Murine Atrial Myocytes

Detailed methods are given in the Supplemental Material.

### Langendorff Heart Preparations for Atrial Electrophysiology

To achieve operator blinding to genotype, mice of equal sex ratio were collected randomly from their cohort by an operator not present during the Langendorff protocol. Genotype was only revealed after complete data analysis. Mice were killed via cervical dislocation, hearts excised and cannulated via the aorta before being mounted onto a modified Langendorff setup. Hearts were retrogradely perfused with 37°C Krebs solution at 2 mL/min containing 118 mmol/L NaCl, 4.75 mmol/L KCl, 1.19 mmol/L MgSO_4_.7H_2_O, 25 mmol/L NaHCO_3_, 1.19 mmol/L KH_2_PO_4_, 5 mmol/L D-glucose, 1.4 mmol/L CaCl_2_, and 2 mmol/L C_3_H_3_NaO_3_, bubbled with 95% O_2_/5% CO_2_. Hypoxia was produced by bubbling Krebs with 95% N_2_/5% CO_2_.

A commercially available flexible microelectrode array ([FlexMEA], Multichannel Systems, Germany) was placed on the surface of the left atrial appendage allowing for measurement of field potentials through accompanying software (MC Rack v4.6.2, Multichannel Systems, Germany). A custom-made unipolar platinum electrode was positioned on the posterior aspect of the heart directly adjacent to the left atrium. Experimental protocols were commenced after a 10-minute equilibration and monitoring period.

Pacing was performed via 1 millisecond biphasic 2 V stimuli using a stimulus generator (STG4002, Multichannel Systems) programmed using a dedicated software interface (MC_Stimulus II, Multichannel Systems, Germany). The programmed electrical stimulation (PES) protocol consisted of a drive train of 8 paced beats (S1) at cycle length (CL) 120 milliseconds each followed by an extra stimulus (S2) every ninth beat. After the first drive train, the S1 to S2 interval was set at 118 milliseconds and subsequently reduced by 2 milliseconds for each subsequent drive train until refractoriness was reached. ERP was taken as 1 milliseconds longer than the shortest coupled S2 that did not activate the tissue. The PES protocol was performed at baseline and repeated at 2-minute intervals of a 20-minute experimental process (12 minutes of hypoxia followed by 8 minutes washout with control perfusate).

Electrograms were analyzed for conduction velocity (CV) using semiautomated custom software running in Matlab (The Mathworks Inc, MA) via a previously validated method.^15^ Briefly, during pacing, the time from the pacing artifact to dV/dt_min_ was used as the local activation time. Electrograms were manually inspected to ensure quality. Grossly fragmented electrograms where no clear dominant negative intrinsicoid deflection of the field potential could be visualized were excluded from the analysis. For steady-state measurements, the number of electrograms was rationalized to those of the first 2 drive trains of the protocol. A topographical function fitted a 2D least-squares regression surface to activation time data across the entire electrode array grid for each beat. The distance between each electrode is known (300 µm) and given the grid nature of the FlexMEA, it is equal in both the *x* and *y* planes. Activation gradients (time/distance) could be measured at each point of the fitted surface corresponding to each included electrode data point. The gradient (G) has vector directionality and at each point is calculated in a Cartesian fashion whereby G = √ (G*x*^2^ + G*y*^2^). The inverse of the activation gradient 1/G is the CV at that point. The mean of these was then taken as the overall CV across the FlexMEA for that beat. The mean was then taken of data from all beats pertaining to a particular coupling interval.

### Statistical Analyses

The data are presented as mean±SEM where residual distribution was tested for normality using the Shapiro-Wilk normality test. The data were analyzed using Microsoft Excel and GraphPad Prism (GraphPad Software Inc., California). Student *t* test and ANOVA were used to compare means where appropriate, with Tukey post hoc test when comparing each mean to every other mean, Dunnett post hoc test when comparing each mean to a control mean, and Bonferroni post hoc test when comparing selected means. Fisher exact test was used to compare proportions of categories in 2 groups. *P<*0.05 was taken to be significant.

## RESULTS

### Differential K_ATP_ RNA Expression in Murine and Human Hearts

**Figure 1.**
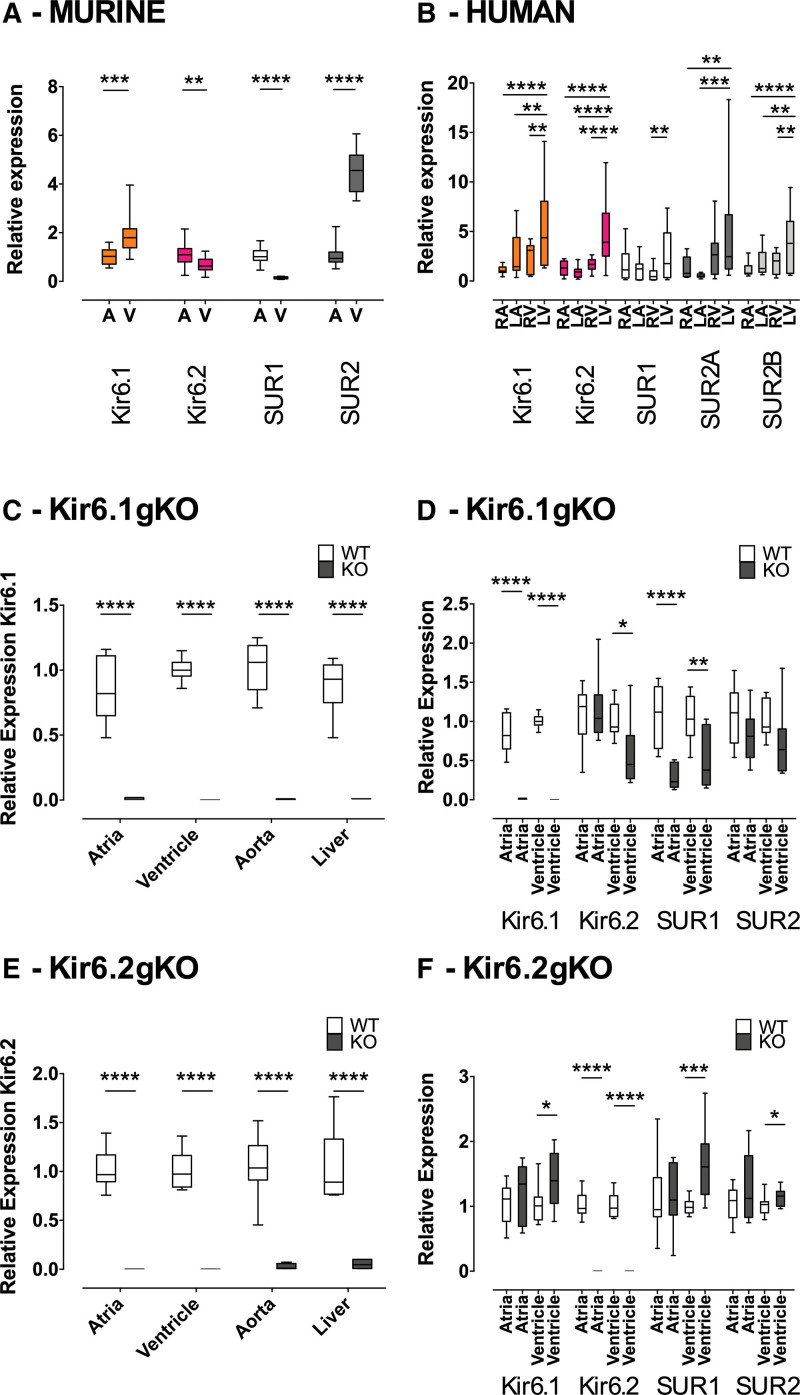
**Cardiac ATP-sensitive potassium channel (K_ATP_) subunit RNA expression.** Differential K_ATP_ subunit expression in (**A**) WT mice (n=16) and (**B**) human (data are from 3 commercially available male donors aged 49, 65, and 69 years all with normal cardiac structure and function and no medical history). Data are normalized to GAPDH and relative to atria (mice) and right atrium ([RA]; human). **C**, Levels of expression of Kir6.1 (*Kcnj8*) in Kir6.1 global knockout (6.1-gKO) relative to wild-type (WT) mice (n=3 both groups) and (**D**) of all K_ATP_ subunits in hearts of 6.1-gKO mice relative to WT (n=3 both groups). **E**, Levels of expression of Kir6.2 (*Kcnj11*) in Kir6.2 global knockout (6.2-gKO) relative to WT mice (n=4 both groups) and (**F**) of all K_ATP_ subunits in hearts of 6.2-gKO mice relative to WT (n=4 both groups). The data are shown as median, interquartile range, 5 to 95 percentile, by (**A** and **B**) 2-way ANOVA with Bonferroni posttest, (**C** through **F**) independent samples *t* test. *P<0.05, **P<0.01, ***P<0.001, ****P<0.0001. A indicates atria; LA, left atrium; LV, left ventricle; RV, right ventricle; and V, ventricles.

Kir6.2 and SUR1 are crucial for the formation of sarcolemmal K_ATP_ channels in murine atrial myocytes (AMs).^6^ An area of interest is whether Kir6.1 also contributes to an AM K_ATP_ current. Wild-type hearts (n=16) were subjected to quantitative reverse transcription polymerase chain reaction. There was a ≈2-fold greater expression of Kir6.1 (*Kcnj8*) transcripts in ventricle compared with atria (*P<*0.001). The opposite was true of Kir6.2 (*Kcnj11*) transcripts where there was a ≈2-fold greater expression in atria compared with ventricle (*P<*0.01; Figure 1A). SUR subunit transcripts were also differentially expressed between the cardiac chambers where in atria, there was a ≈7-fold greater expression of SUR1 (*Abcc8*) compared with ventricle (*P<*0.0001), while SUR2 (*Abcc9*) was expressed ≈4-fold more in ventricle compared with atria (*P<*0.0001; Figure 1A).

K_ATP_ subunit transcript expression was then tested in human heart. Whole heart RNA from all 4 chambers from 3 commercially available male donors aged 49, 65, and 69 years all with normal cardiac structure and function and no past medical history were subjected to quantitative reverse transcription polymerase chain reaction (Figure 1B). In contrast to murine heart, there was an equal expression of all K_ATP_ subunit RNA across right atrium, left atrium, and right ventricle, but increased expression in left ventricle compared with the other chambers. This met statistical significance for SUR1 (ABCC8) only between left ventricle and right ventricle and SUR2A (*ABCC9*) only between left ventricle and each atrium.

### Characterization of RNA Expression in Kir6.1 and Kir6.2 Knockout Mice

We explored further the contribution of Kir6.1 to a K_ATP_ current in isolated murine AMs using Kir6.1 global knockout (6.1-gKO) and Kir6.2 global knockout (6.2-gKO) mice. To confirm the global deletion of Kir6.1 and Kir6.2 in knockout mice, quantitative reverse transcription polymerase chain reaction was used on total RNA isolated from atria, ventricles, aorta, and liver of global knockouts and their littermate controls. In both 6.1-gKO and 6.2-gKO mice, there was complete deletion of the respective pore-forming subunit transcripts (*P<*0.0001; Figure 1C and 1E). Interestingly, Kir6.2 transcript expression was reduced by ≈50% in the ventricles of 6.1-gKO mice (*P<*0.05) and SUR1 transcript expression reduced by a similar degree in both the atria (*P<*0.0001) and ventricles (*P<*0.01) of these mice (Figure 1D). In the ventricles of 6.2-gKO mice, Kir6.1, SUR1, and SUR2 transcript expression was increased by ≈40% (*P<*0.05), ≈60% (*P<*0.001), and ≈15% (*P<*0.05), respectively (Figure 1F).

### Electrophysiological Characterization of Isolated AMs in Kir6.1 and Kir6.2 Knockout Mice

**Figure 2.**
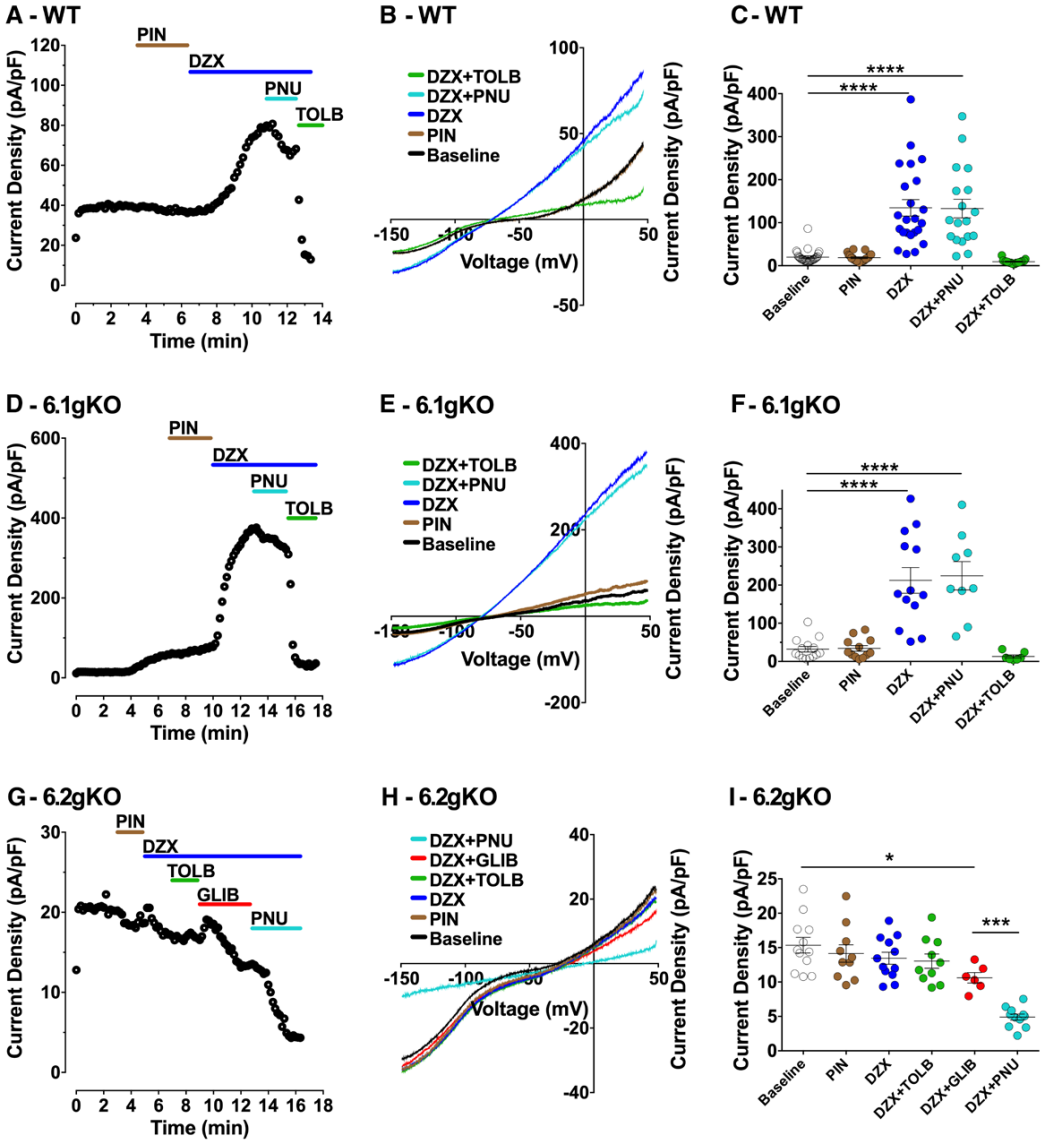
**ATP-sensitive potassium channel (K_ATP_) current in isolated murine atrial cardiomyocytes. A**, **D**, and **G**, representative time-course traces at +40 mV; **B**, **E**, and **H**, whole-cell current density-voltage traces; **C**, **F**, and **I**, summary of mean whole-cell current densities at +40 mV in isolated atrial myocytes (AMs) from wild type (WT), Kir6.1 global knockout (6.1-gKO), and Kir6.2 global knockout (6.2-gKO) mice. Current density-voltage relationships were recorded using a 1-second ramp protocol (−150 mV to +50 mV) from a holding potential of −80 mV. Summary data are shown as mean±SEM (WT, n=17–28 cells from 13 mice; 6.1-gKO, n=8-14 cells from 4 mice; 6.2-gKO, n=6-12 cells from 5 mice), by 1-way ANOVA with Dunnett posttest vs baseline and Bonferroni posttest for selected means. *P<0.05, ***P<0.001, ****P<0.0001. DZX indicates diazoxide 100 µmol/L; GLIB, glibenclamide 10 µmol/L; PIN, pinacidil 10 µmol/L; PNU, PNU37883 50 µmol/L; and TOLB, tolbutamide 100 µmol/L.

The pharmacological fingerprint of K_ATP_ channels of all homomultimeric Kir6x/SURx combinations was assessed using whole-cell patch clamp in a HEK293 cell stable overexpression system (see Figures S1 and S2). This pharmacology was then applied to pooled (right and left) murine AMs. In wild-type (WT) AMs (n=17–28 cells/13 mice), a current could be activated by diazoxide 100 µmol/L ([DZX]; *P<*0.0001 versus baseline) but not pinacidil 10 µmol/L (Figure 2A through 2C). This current was inhibited subsequently by tolbutamide 100 µmol/L ([TOLB]; *P*<0.0001 versus DZX; *P*>0.05 versus baseline) but not PNU37883 50 µmol/L ([PNU]; *P>*0.05 versus DZX; *P<0*.0001 versus baseline), all in keeping with a Kir6.2/SUR1 current. The same pharmacological fingerprint was true for 6.1-gKO murine AMs (n=8–14 cells/4 mice) initially suggesting little or no contribution of Kir6.1 to a K_ATP_ current in these cells (Figure 2D through 2F).

In 6.2-gKO murine AMs, both DZX and pinacidil 10 µmol/L failed to activate a current (n=6-12 cells/5 mice; Figure 2G through 2I). However, there was a small baseline current, ≈5 pA/pF, sensitive to glibenclamide 10 µmol/L ([GLIB]; *P<*0.05 versus baseline) and there was a further ≈5 pA/pF, sensitive to subsequent application of PNU (*P<*0.001 versus GLIB). The inference being that a small Kir6.1-containing K_ATP_ current existed after deletion of Kir6.2.

**Figure 3.**
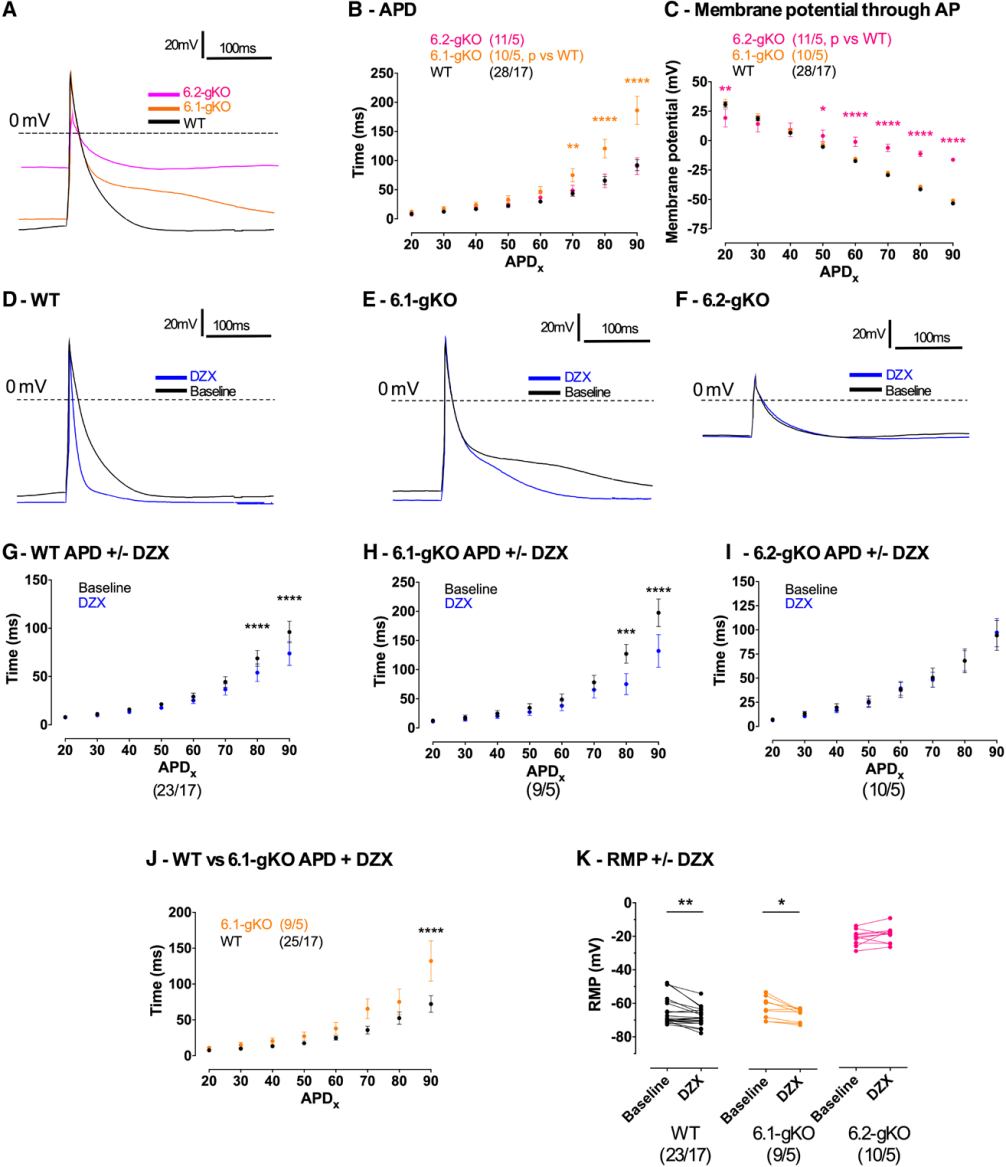
**Action potentials (APs) in isolated murine atrial cardiomyocytes. A**, Overlay of wild type (WT), Kir6.1 global knockout (6.1-gKO), and Kir6.2 global knockout (6.2-gKO) APs at baseline. **B**, AP duration at various percentage phase of repolarization (APDx). **C**, Membrane potential at various percentage phase of repolarization (APDx). **D** through **F**, Representative APs at baseline and with application of the ATP-sensitive potassium channel (K_ATP_) activator diazoxide 100 µmol/L (DZX). **G** through **J**, Effects of DZX on APD. **K**, Effect of DZX on resting membrane potential in atrial myocytes (AMs) from WT, 6.1-gKO and 6.2-gKO mice. Whole-cell current-clamp recordings were made to 600 pA/5 milliseconds stimulating square pulses at 1 Hz. Data are shown as mean±SEM (n cells/n mice), by (**B** and **C**) repeated measures 2-way ANOVA with Dunnett posttest vs WT, (**G** through **I**) repeated measures 2-way ANOVA with Bonferroni posttest, (**J**) 2-way ANOVA with Bonferroni posttest, (**K**) paired *t* test. *P<0.05, **P<0.01, ***P<0.001, ****P<0.0001.

To further explore the influence of K_ATP_ on murine AM electrophysiology, we recorded APs in isolated murine AMs. Isolated AMs from 6.2-gKO mice were more depolarized (*P<*0.0001) and APs had slower upstrokes (*P<*0.0001) and smaller amplitudes compared with WT and 6.1-gKO mice (*P<*0.0001; Table; Figure 3A). Under the conditions of our study, we failed to measure a resting membrane potential (RMP) more negative than ≈−30 mV in AMs isolated from 6.2-gKO mice.

**Table.**
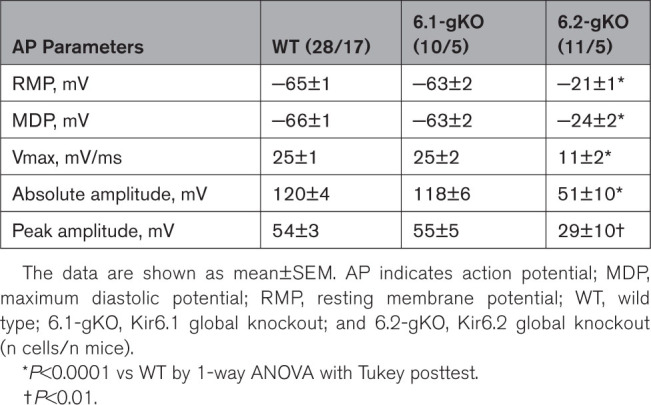
Summary of Action Potential Parameters

Baseline AM action potential duration (APD) was no different to WT for 6.2-gKO mice; however, 6.1-gKO mice AMs had longer APs from 70% to 90% repolarization (Figure 3A and 3B). Owing to effects on RMP and amplitudes of the 6.2-gKO mice AM AP, but no effects in these mice on the APD, this translated to differences in the membrane potential at any phase of repolarization. As such beyond APD_50_, membrane potential in AMs of 6.2-gKO mice was always more depolarized (Figure 3C). No such differences were seen in 6.1-gKO mice.

DZX shortened APD, particularly beyond 70% repolarization, in WT and 6.1-gKO AMs but failed to affect APD in 6.2-gKO AMs (Figure 3D through 3I). Despite DZX, APD remained longer at 90% repolarization in 6.1-gKO AMs compared with WT (*P<*0.0001; Figure 3J). DZX hyperpolarized RMP in WT (*P<*0.01) and 6.1-gKO AMs (*P<*0.05) but 6.2-gKO AMs failed to respond (Figure 3K).

Despite the effect of an increased afterload and coronary spasm seen previously in Kir6.1 knockout,^16^ and cardiac hypertrophy/heart failure phenotypes described in Kir6.2 knockout animals,^17^ there were no differences in cell capacitance (surrogate for cell size) of AMs from WT, 6.1-gKO, and 6.2-gKO mice at this age (Figure S3).

Expression of a host of ion channel, hypertrophy marker, and fibrosis marker genes was then assessed in both atria and ventricle from 6.1-gKO mouse hearts and compared with WT. This revealed only one difference namely increased expression of the fibrosis marker gene *Ctgf* in the atria (see Figure S4). However, we then performed histological analysis in all genotypes and found no signs of replacement fibrosis or myocyte loss in the atria or ventricle of 6.1-gKO or 6.2-gKO mouse hearts (see Figure S5).

In summary, knockout of Kir6.1 leads to more prolonged isolated AM APs compared with WT. Absence of Kir6.2 led to isolated AM APs that were more depolarized.

### Knockout of Both K_ATP_ Pore-Forming Subunits Leads to Functional Changes in Murine Atrial Tissue Electrophysiology

**Figure 4.**
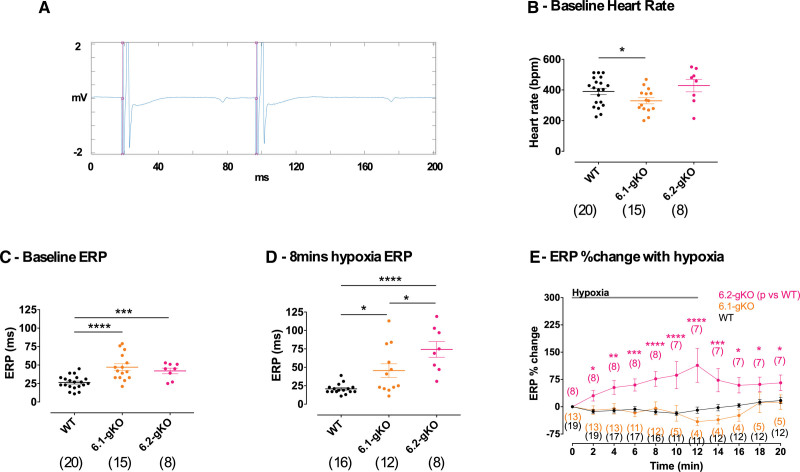
**Atrial effective refractory period (ERP) in Langendorff mouse hearts. A**, Example signal window showing final S1 and S2 beats of a drive train. The pink lines show timing of automatically detected pacing artifact followed by the induced atrial activation and electrogram. Note can be made of the large sharp local atrial signal and small far-field ventricular electrogram. **B**, Baseline sinus heart rate. **C**, Baseline ERP. **D**, ERP at 8 minutes of hypoxia. **E**, ERP change normalized to baseline during hypoxia. Data are shown as mean±SEM (n), by (**B** through **D**) 1-way ANOVA with Tukey posttest, (**E**) 2-way ANOVA with Dunnett posttest vs WT. *P<0.05, **P<0.01, ***P<0.001, ****P<0.0001. 6.1-gKO indicates Kir6.1 global knockout; and 6.2-gKO, Kir6.2 global knockout.

We next investigated whether the effects on K_ATP_ current density and AP morphology seen in isolated murine AMs translated to tissue level. In Langendorff-perfused hearts, interval measurements were made of ERP and CV at baseline, during a 12-minute period of hypoxia to metabolically activate a K_ATP_ current and an 8-minute washout period. In some hearts, TOLB was also perfused during 8 to 12 minutes of hypoxia. Example electrogram data acquired from the FlexMEA are shown in Figure 4A, where note can be made of the sharp local atrial signal of large amplitude, and the lower frequency, small amplitude far-field ventricular signal.

Baseline sinus node firing rate was ≈60 bpm slower in 6.1-gKO (n=15 mice; *P<*0.05) mice compared with WT (n=20 mice; Figure 4B; Table S1). Baseline atrial ERP was ≈80% longer in 6.1-gKO (n=15 mice; *P<*0.0001) and ≈60% longer in 6.2-gKO (n=8 mice; *P<*0.001) compared with WT (n=20 mice; Figure 4C; Table S1). ERP was maintained during the hypoxia-reperfusion protocol in WT and 6.1-gKO mice normalized to baseline but had nearly doubled by 12 minutes of hypoxia normalized to baseline in 6.2-gKO mice (*P<*0.0001), before returning toward baseline with reperfusion of oxygenated solution (Figure 4D and 4E; Table S1). Application of TOLB during 8 to 12 minutes of hypoxia in WT and 6.1-gKO mice induced prolongation of ERP normalized to baseline akin to that seen during hypoxia in 6.2-gKO mice not treated with TOLB (see Figure S6A and S6B; Table S1).

**Figure 5.**
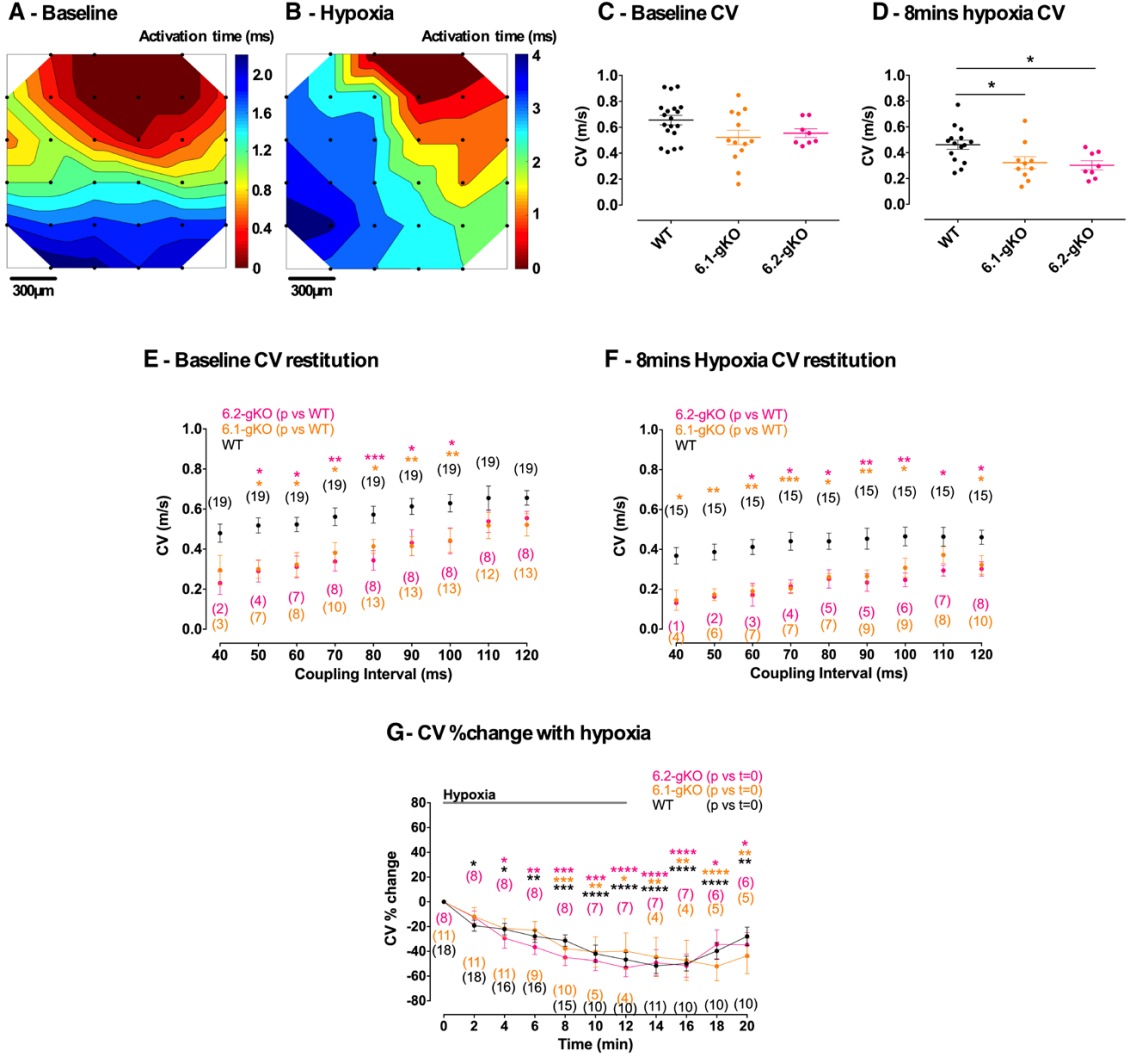
**Atrial conduction velocity (CV) in Langendorff mouse hearts. A** and **B**, Example activation maps across the flexible microelectrode array (FlexMEA) covering the surface of the left atrial appendage. Black dots denote active electrodes and contours and color fill signify isochrones of activation. Activation times are given by the color bar legend. **A**, Baseline and (**B**) hypoxia demonstrating conduction slowing. Stimulation is adjacent to the top right electrode in each case. (**C**) Baseline steady-state CV (120 milliseconds coupling interval). **D**, Steady-state CV at 8 minutes of hypoxia. **E**, CV restitution at baseline. **F**, CV restitution at 8 minutes of hypoxia (steady state = 120 milliseconds coupling interval). **G**, CV change normalized to baseline during hypoxia. Data are shown as mean±SEM (n), by **C** and **D** 1-way ANOVA with Tukey posttest, (**E** through **G**) 2-way ANOVA with Dunnett posttest vs WT or baseline as indicated. *P<0.05, **P<0.01, ***P<0.001, ****P<0.0001. 6.1-gKO indicates Kir6.1 global knockout; and 6.2-gKO, Kir6.2 global knockout.

The isochronal activation maps in Figure 5A and 5B demonstrate planar conduction across the FlexMEA and slower conduction under hypoxic conditions. A trend toward a reduction in atrial baseline steady-state CV was seen in both pore subunit knockout hearts compared with WT (Figure 5C), which became ≈40% slower (*P<*0.05) with restitution at shorter coupling intervals (Figure 5E; Table S2). Steady-state CV was ≈20% slower (*P<*0.05) in both knockouts compared with WT during hypoxia (Figure 5D), and this was accentuated with restitution at shorter coupling intervals (Figure 5F; Table S2). Steady-state CV progressively reduced normalized to baseline during hypoxia in all genotypes and there were no effects of TOLB application during hypoxia in WT and 6.1-gKO mice or on CV restitution (Figure 5G; Figure S6C through S6E; Table S2).

**Figure 6.**
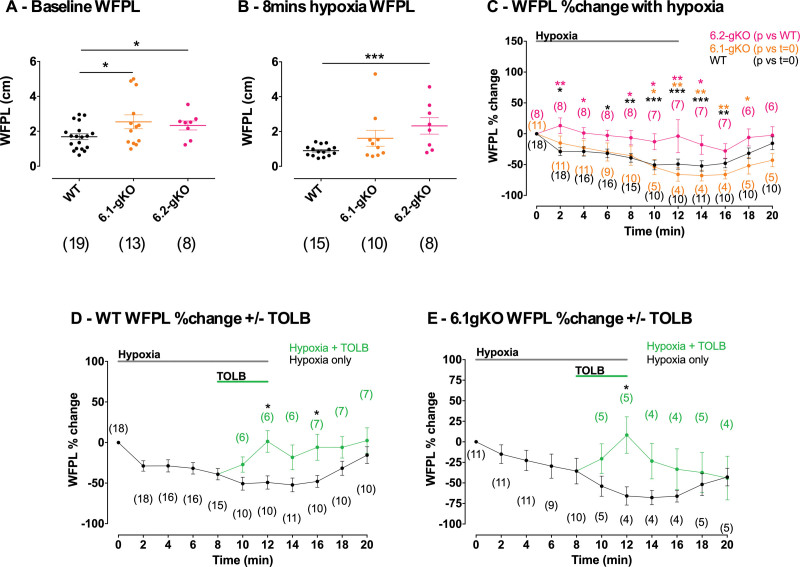
**Atrial minimum wavefront path length (WFPL) in Langendorff mouse hearts. A**, Baseline WFPL. **B**, WFPL at 8 minutes hypoxia. **C**, WFPL change normalized to baseline during hypoxia. **D**, WFPL change normalized to baseline during hypoxia with and without tolbutamide 100 µmol/L (TOLB) ATP-sensitive potassium channel (K_ATP_) inhibition in wild-type (WT) mice. **E**, As for (**D**) but for Kir6.1 global knockout (6.1-gKO) mice. Data are shown as mean±SEM (n), by (**A** and **B**) 1-way ANOVA with Tukey posttest, (**C**) 2-way ANOVA with Dunnett posttest vs WT or baseline as indicated, (**D** and **E**) 2-way ANOVA with Bonferroni posttest. *P<0.05, **P<0.01, ***P<0.001. 6.2-gKO indicates Kir6.2 global knockout.

Atrial wavefront path length (WFPL) was then calculated as the product of the ERP and steady-state CV. Baseline WFPL was ≈50% and ≈35% longer in 6.1-gKO (n=13 mice; *P<*0.05) and 6.2-gKO (n=8 mice; *P<*0.05), respectively, compared with WT (n=19; Figure 6A; Table S3). Twelve minutes of hypoxia shortened WFPL to ≈50% of baseline in WT mice (*P<*0.001) and ≈70% of baseline in 6.1-gKO mice (*P<*0.01) but was maintained normalized to baseline in 6.2-gKO mice (Figure 6B and 6C; Table S3). Application of TOLB during 8 to 12 minutes of hypoxia in WT and 6.1-gKO mice induced prolongation of WFPL back toward baseline measurements, akin to the maintenance of WFPL seen during hypoxia in 6.2-gKO mice not treated with TOLB (Figure 6D and 6E; Table S3).

In summary, both K_ATP_ pore subunits contribute to electrophysiological parameters of atrial tissue both at baseline and during hypoxia-ischemia, although Kir6.2 had most influence.

### 6.2-gKO Mice Display Reduced Arrhythmia Inducibility to PES During Hypoxia-Ischemia

**Figure 7.**
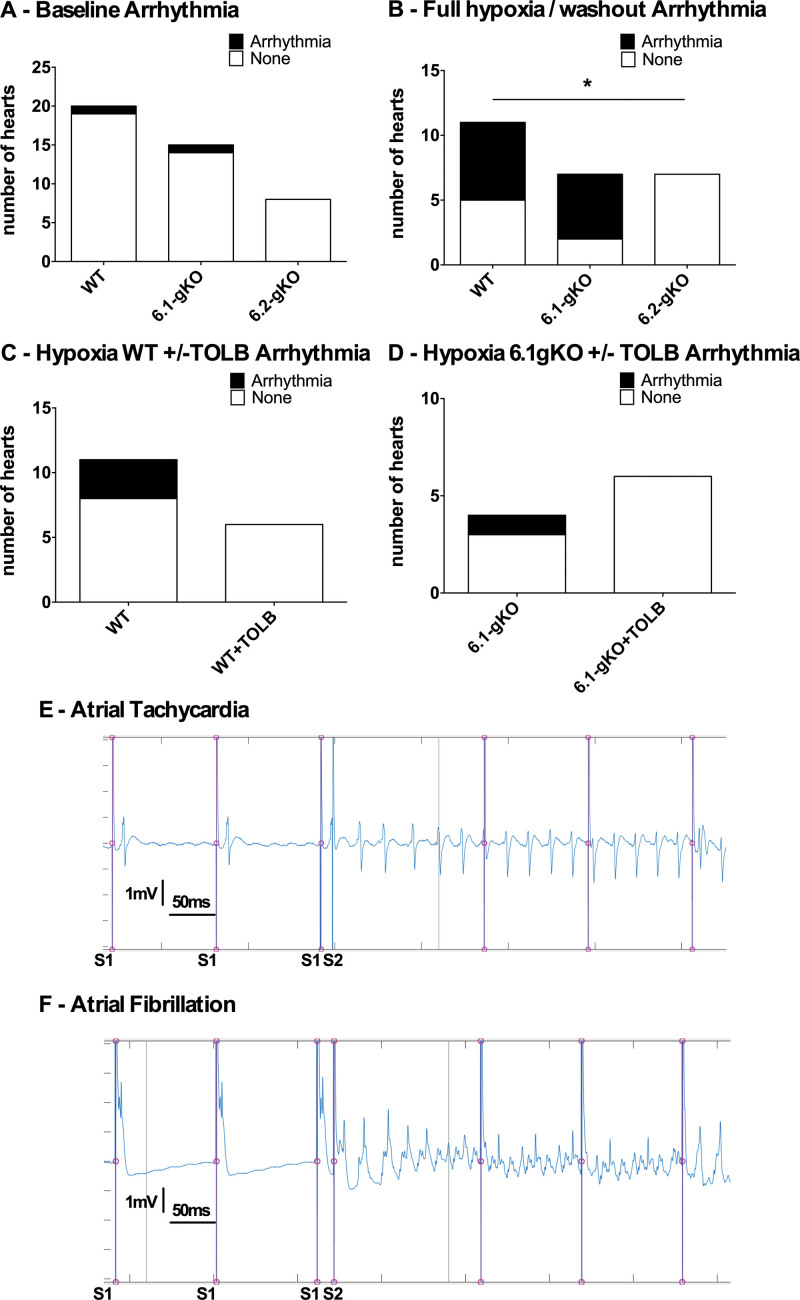
**Kir6.2 global knockout (6.2-gKO) mice atria display reduced arrhythmia inducibility to programmed electrical stimulation (PES) during hypoxia/reperfusion.** Proportions of hearts with PES-induced sustained atrial arrhythmia (500 milliseconds or longer): (**A**) At baseline; (**B**) during the hypoxia/washout 20-minute protocol; (**C**) 8 to 12 minutes of hypoxia with and without ATP-sensitive potassium channel (K_ATP_) inhibition in the wild type (WT); (**D**) as (**C**) but for Kir6.1 global knockout (6.1-gKO). **E**, Example trace of arrhythmia induced by programmed electrical stimulation. Trace showing the final 3 beats of a drive train S1 with preceding pacing artifact, followed by the S2 with shorter coupling interval also with preceding pacing artifact, followed by atrial tachycardia (further pacing artifact is also present from the next drive train in the automated sequence), which on this occasion went on to last for 25 beats and ≈700 milliseconds. **F**, As for (**E**) but example trace of induced atrial fibrillation, which went on to last for ≈2 seconds. *P<0.05 by Fisher exact test. TOLB indicates tolbutamide 100 µmol/L.

Tissue properties allowing for a shorter WFPL will increase the likelihood of reentrant arrhythmias.^18^ Further to the differences in atrial WFPL between genotypes seen in response to hypoxia, particularly shortening of atrial WFPL with hypoxia in WT and 6.1-gKO mice, but maintenance of atrial WFPL in 6.2-gKO mice, we then sought to establish if this translated into a reduced ability to induce atrial arrhythmias in 6.2-gKO mice. Arrhythmias reproducibly induced with PES tend to have a reentrant mechanism.^19^ We used a cutoff for sustained arrhythmia of 500 milliseconds or longer, and investigated the proportion of hearts with sustained atrial arrhythmia induced after S2 of a drive train at baseline, and throughout the hypoxic protocol. While at baseline atrial WFPL was prolonged compared with WT in 6.1-gKO and 6.2-gKO mice, atrial arrhythmia was induced in only 1 of 20 WT hearts, 1 of 15 6.1-gKO hearts, and 0 of 8 6.2-gKO hearts. During hypoxia, however, atrial arrhythmia was induced in 6 of 11 WT hearts, 5 of 7 6.1-gKO hearts, and 0 of 7 6.2-gKO hearts (Figure 7A and 7B: 6.2-gKO versus WT during protocol; *P<*0.05 by Fisher exact test). Further, these arrhythmias were all induced in the hypoxic period up to 16 minutes of the protocol, corresponding to the period where the WFPL differed significantly between the 6.2-gKO and WT and 6.1-gKO mice due to WFPL shortening in the latter 2 genotypes (Figure 6C).

We then assessed arrhythmia inducibility during the 8- to 12-minute period of the protocol where in a proportion of WT and 6.1-gKO hearts TOLB was applied during hypoxia, which led to WFPL prolongation (Figure 6D and 6E). While arrhythmias were inducible in the absence of TOLB (Figure 7C and 7D: WT 3/11 hearts, 6.1-gKO 1/4 hearts), no arrhythmias were inducible in the presence of TOLB (Figure 7C and 7D: WT+TOLB 0/6 hearts; 6.1-gKO+TOLB 0/6 hearts).

In total, 37 episodes of arrhythmia were induced, where 15 were atrial tachycardia and 22 were atrial fibrillation (Figure 7E and 7F). No spontaneous arrhythmias were witnessed in the 10-minute equilibration and monitoring period before the PES and hypoxia/washout protocols were commenced.

## DISCUSSION

We have examined the role of K_ATP_ channels in determining atrial electrophysiology at the cellular and tissue level. Novel findings are that first, during hypoxia, K_ATP_ activation leads to maintenance of atrial ERP in the intact heart. Maintenance of ERP in murine atria is dependent on Kir6.2 as in 6.2-gKO mice ERP prolongation accompanied conduction slowing during hypoxia. This meant WFPL was maintained during hypoxia and absence of arrhythmia inducibility to PES. In WT and 6.1-gKO mice, conduction slowing occurred with hypoxia but with maintenance of ERP this led to a critical shortening of WFPL that was accompanied by atrial arrhythmia inducibility. Thus, activation of Kir6.2-containing K_ATP_ during hypoxia in murine atria is proarrhythmic. Second, Kir6.1 contributes to an atrial sarcolemmal K_ATP_ current, although Kir6.2, in keeping with previous reports, is the predominant pore-forming subunit in murine AMs.

### Effects of K_ATP_ Pore Knockout on Atrial ERP and CV

Under the conditions of this study, isolated AMs were reproducibly more depolarized in the absence of Kir6.2 with reduced V_max_ and peak amplitude; 6.1-gKO AM APD was significantly longer. We did not see evidence for remodeling of other ionic currents based on their RNA expression. Earlier studies of Kir6.2 knockout mice did not show baseline changes in RMP or APD in the ventricle.^20,21^ Baseline atrial APD was longer in 1 study in Kir6.2 knockout mice compared with WT and no different in another study, although membrane voltages were not discussed in these studies^12,13^; 6.2-gKO atrial APD also did not differ in our study. All of the prior studies were conducted solely in the intact heart via transmembrane APs or optical APs. Isolated APs have been studied in ventricular myocytes of Kir6.2 knockout mice with no difference of baseline AP parameters.^22^ To our knowledge, there have been no prior studies of APs in isolated AMs comparing WT to Kir6.1 and Kir6.2 knockout animals. Electrotonic effects of cell-to-cell coupling are lost in isolated cells. The RMP of isolated WT AMs in this study are comparable to those of other studies.^23,24^ The isolated AMs in our study may be more depolarized than in the intact heart, and there may be a background activation of K_ATP_ due to stress after cell isolation. It is worth noting that it has already been demonstrated that SUR1-containing K_ATP_ channels in isolated murine AMs activate more readily even in the absence of metabolic inhibition, and that this is in contrast to the ventricle.^6,25^ This implies K_ATP_ deletion would lead to depolarizing effects on baseline RMP in AMs. The isolated AMs of 6.2-gKO may overestimate the degree of depolarization, and we recognize this as a potential limitation of the study of isolated AMs. However, the implication that K_ATP_ is active and contributes to cardiomyocyte RMP and APD under baseline conditions, while controversial has been suggested before.^6,12,25–27^

Our findings are also qualitatively consistent with our observations in the intact heart. Depolarization in 6.2-gKO AMs would lead to reduced sodium channel availability due to inactivation at more depolarized membrane potentials, and a greater reliance on the inward calcium current.^28^ This would lead to the reduced V_max_ and reduced peak amplitude at baseline in isolated 6.2-gKO AM APs that we observed, and the slowing of CV in tissue with restitution at shorter coupling intervals we observed in the intact heart. No histological evidence of replacement fibrosis or myocyte loss was observed. ERP prolongation at baseline in the 6.2-gKO intact heart likely results from a mechanism akin to postrepolarization refractoriness again due to reduced sodium channel availability.^29^

Longer ERP at baseline in the 6.1-gKO intact atria can be explained by a longer APD observed in the isolated AMs. Evidence exists that K_ATP_ activation provides repolarization adaptation to higher heart rates during stress.^20^ 6.1-gKO APD maladaptation can be expected to cause slowing of CV particularly at shorter coupling intervals, such as rapid pacing, where even small degrees of membrane potential depolarization can slow the kinetics of recovery from inactivation of sodium channels.^28^

### K_ATP_ Channels and Ischemically Driven Atrial Arrhythmias

K_ATP_ channels are activated by ischemia: increases of as little as 0.7% of maximal K_ATP_ conductance lead to a 50% reduction in APD.^30^ Subsequent reductions in ventricular ERP have been shown to lead to an increase in arrhythmia inducibility that is reversed with K_ATP_ inhibition.^8^ While models have shown K_ATP_ activation in the atria leads to increased arrhythmia inducibility, this was driven by adrenergic drive or K_ATP_ activating drugs at high doses, and not by hypoxia/ischemia.^7,9,10^ In a murine model exploring knockout of Kir6.2 and SUR1, a failure of AP and calcium transient shortening led to focal ventricular activity indicative of afterdepolarizations, although this was induced with metabolic poisoning rather than hypoxia/ischemia.^13^ In the current study, we used PES to initiate reentrant arrhythmias. Hypoxia reduces membrane currents and transporter activity in cardiomyocytes, and as a result the RMP becomes more depolarized, the AP amplitude is reduced, and conduction in tissue slows.^31^ Our study has shown K_ATP_ activation provides compensation for the effects of hypoxia and in the murine atria Kir6.2 is the predominant pore-forming subunit providing this effect. Unlike in WT and 6.1-gKO mice, APD failed to shorten with application of DZX in isolated AMs from 6.2-gKO mice and this is likely to translate to a failure of AP shortening during hypoxia. Our findings in the atria were also demonstrated in the ventricle in another study investigating a murine Kir6.2 knockout model, where there was no APD shortening in response to ischemia.^21^ In our study, Kir6.2 was able to compensate for Kir6.1 deletion by maintaining the ERP in 6.1-gKO atria during hypoxia compared with baseline, albeit the absolute ERP was longer than WT akin to the longer APD with DZX in isolated AMs. Prolongation of ERP despite conduction slowing during hypoxia in 6.2-gKO mice meant that WFPL was maintained similar to observations in the ventricle by another group.^21^

Arrhythmias induced with PES are widely assumed to be reentrant in mechanism. WFPL maintenance during hypoxia in the 6.2-gKO mice, and as a corollary maintenance of the excitable gap, correlated with an absence of arrhythmia inducibility to PES that was observed in WT and 6.1-gKO mice where WFPL shortened. Tolbutamide block of the active Kir6.2-containing K_ATP_ population during hypoxia in WT and 6.1-gKO mice led to WFPL lengthening.

Atrial ischemia during cardiac surgery and distribution of occlusive coronary disease involving atrial branches are associated with an increased propensity to postoperative atrial fibrillation.^32^ Coronary artery disease affecting the atrial branches is an independent predictor for the development of AF after myocardial infarction.^33^ Atrial ischemia promotes atrial fibrillation in dogs seemingly through conduction slowing rather than ERP shortening.^34^ This effect, and more specifically an increased heterogeneity of CV, was replicated in sheep.^2^ Atrial ERP did shorten secondary to atrial ischemia in another canine study but this was not reversed by application of the K_ATP_ inhibitor GLIB.^35^

The electrophysiological effects of hypoxia/ischemia on the atria, as in the ventricles, can be expected to be heterogeneous across the myocardium.^2^ If K_ATP_ opening serves to counter the effects of membrane depolarization and maintain ERP during hypoxia/ischemia, the increase in variability of atrial ERP with hypoxia/ischemia after K_ATP_ pore knockout as seen in this study is not unexpected.

Our study supports the effect of K_ATP_ opening to maintain the ERP during hypoxia, and together with conduction slowing this leads to WFPL shortening. This provides a mechanism for an increased arrhythmogenic substrate for reentry during hypoxia-ischemia.

### Atrial Sarcolemmal Kir6.1 and Chamber-Specific K_ATP_ Channel Pharmacology

Prior studies support the presence of a population of sarcolemmal Kir6.1-containing channels in rodent AMs. These include an intermediate conductance channel in rat AMs, and differential basal activity of rat atrial versus ventricular cardiomyocytes in the presence of 84 µM ADP.^25,36^ Kir6.1-containing K_ATP_ channels are sensitive to activation by MgADP and less sensitive to direct ATP inhibition than Kir6.2-containing K_ATP_ channels.^37^ In 6.2-gKO mice AMs, the K_ATP_ current was almost completely eliminated, although a small but measurable 5 pA/pF current remained that was inhibited by GLIB, a selective K_ATP_ inhibitor. A further 5 pA/pF current was inhibited by PNU but we also saw this in untransfected HEK293 cells where the GLIB effect was not witnessed, and this may represent off-target effects of PNU (see Supplemental Material). There was an inability to activate current from baseline in 6.2-gKO mice, unlike in the WT and 6.1-gKO mice. We postulate that a remaining fully activated Kir6.1 current was present at baseline due to sensitivity to ADP in the pipette solution. This is a recognized property of Kir6.1-containing channels and basally active Kir6.1-containing channels have been identified at the cardiomyocyte sarcolemma previously.^27,38^ There was no apparent reduction in K_ATP_ current in isolated AMs from 6.1-gKO mice. Kir6.2 is the predominant atrial pore and has a larger unitary conductance.^36^ This may have prohibited unmasking the change in whole-cell current in these mice.

6.1-gKO AM APD remained longer after the application of DZX and 6.1-gKO murine atria continued to exhibit a longer absolute ERP and slower CV than WT mice during hypoxia. Knockout of Kir6.1 in vascular smooth muscle leads to hypertensive animals,^16^ but we did not see an increase in cell capacitance indicative of hypertrophy at the study age of animal in these AMs. We also did not observe any difference in the expression of a host of other ion channel, hypertrophy marker, or fibrosis marker genes, barring the sole increase in the fibrosis marker *Ctgf*. However, this was not met with any histological evidence of replacement fibrosis or myocyte loss in the knockout mice.

Initial excitement of a pharmacologically targetable differential SURx subunit composition of K_ATP_ between atria and ventricle in the rodent heart were tempered by the suggestion of a lack of such differential pharmacology in the human heart.^7^ Our dissection of the expression of K_ATP_ mRNA transcripts in the human heart also suggests the relative pattern of expression of subunits across heart chambers did not differ between the subunits. This places an equal emphasis on Kir6.1 to Kir6.2 in human as compared with murine atria. Various studies have demonstrated a high expression of Kir6.1 transcripts in the human heart.^7,39^ There has also been an association between a gain-of-function mutation S422L in *KCNJ8* (encoding Kir6.1) with the human J wave syndromes and atrial fibrillation.^40^

### Clinical Implications

Pharmacological K_ATP_ blockade could be explored to reduce the burden of hypoxia-ischemia driven atrial arrhythmias such as AF. Selective pore-blockers could be pursued given the precedent of PNU and drug development could be spurred by the recent determination of the cryo-EM structure of K_ATP_.^41^ Crucially there may be detrimental sequelae to K_ATP_ inhibition. K_ATP_ channel opening is protective in the process of ischemic preconditioning, although debate exists as to whether this is due to effects on sarcolemmal or mitochondrial K_ATP_ channels.^42^ Loss of K_ATP_ function in Kir6.2 knockout mice has been shown to promote triggered activity in the form of early afterdepolarizations with isoproterenol challenge, and a similar mechanism of otherwise lone AF demonstrated due to a missense mutation in *ABCC9* encoding SUR2A.^43,44^

The sulphonylureas inhibit K_ATP_ by binding at the SUR subunit and are used as antidiabetic drugs.^45^ There are various series commenting on the pro and antiarrhythmic effects in the ventricle of sulphonylureas used in diabetic patients.^46^ There are no data pertaining to the prevalence of atrial arrhythmias among this patient cohort with concomitant sulphonylurea usage.

### Limitations

The electrophysiological changes seen in isolated AMs may not be reflective of physiology in the intact heart due to loss of electrotonic cell-to-cell coupling and the metabolic challenges of cell isolation. Kir6.1 knockout mice are known to be hypertensive and exhibit episodes of coronary spasm,^16^ although we did not see hypertrophy or fibrosis in the atria of these mice. We have postulated that the electrophysiological effects seen in the knockout mice are secondary to direct effects via a sarcolemmal K_ATP_ current, although indirect effects via a mitochondrial K_ATP_ current cannot be excluded. Mice were of equal sex ratio and differences that could be due to sex were not controlled for.

### Conclusions

In conclusion, we show that both pore-forming K_ATP_ subunits contribute to atrial electrophysiology in the mouse heart, and support that both of these subunits make up the pore of a sarcolemmal channel in AMs. K_ATP_ opening during hypoxia helps counter the effects of hypoxia that lead to ERP prolongation and conduction slowing. K_ATP_ blockade during hypoxia leads to a reduced atrial arrhythmogenic propensity due to minimizing the WFPL shortening afforded by K_ATP_ opening. This gives important added insight into the mechanism of ischemically driven atrial arrhythmias such as AF. This could be pursued translationally to explore the antiarrhythmic effects of pharmacological K_ATP_ blockade.

## ARTICLE INFORMATION

### Sources of Funding

This work was supported by the British Heart Foundation (RG/15/15/31742), Medical Research Council (MR/L016230/1), and National Institute of Health Research R-01 (1R01HL146514-01A1).

### Disclosures

None.

### Supplemental Material

Supplemental Methods

Supplemental Results

Tables S1–S3

Figures S1–S9

References 47–49

## Supplementary Material


