# Supplementary material for: Hypoxia Promotes Atrial Tachyarrhythmias via Opening of ATP-Sensitive Potassium Channels
Source: Circ Arrhythm Electrophysiol. 2023 Aug 30;16(9):e011870. doi: 10.1161/CIRCEP.123.011870 (PMC10510820; doi:10.1161/CIRCEP.123.011870)
Supplement: Supplementary file 1 [file hae-16-e011870-s001.pdf]

SUPPLEMENTAL MATERIAL

Supplemental Methods

Molecular biology and cell culture

cDNAs encoding the Kir6x pore-forming subunits (rat Kir6.1, mouse Kir6.2) were propagated in the pcDNA3.1/Zeo(+) mammalian expression vector (Life Technologies Ltd Invitrogen Division, Paisley, UK) and had been previously obtained by the laboratory as a gift from Professor S. Seino (Chiba University School of Medicine, Chiba, Japan). cDNAs encoding the SURx subunits (hamster SUR1, mouse SUR2A and SUR2B) were propagated in the pcDNA3 mammalian expression vector (Life Technologies Ltd Invitrogen Division, Paisley, UK). SUR1 cDNA was a kind gift from Professor J. Bryan (Baylor College of Medicine, Houston, USA). SUR2A and SUR2B cDNAs were a gift from Professor Y. Kurachi (Osaka University, Osaka, Japan).

HEK293 cells (a human embryonic kidney cell line) were cultured in minimal essential medium with Earle's Salts, L-glutamine supplemented with 10% fetal bovine serum, 1% penicillin/streptomycin (from a stock of 10,000 units/ml penicillin and 1 mg/ml streptomycin) at 37 °C in humidified 5% CO_2. Cells were transfected with Lipofectamine (Life Technologies, Inc.) according to the manufacturer's instructions. Stable cell lines were established using appropriate antibiotic selection with 1mmol/L G418 disulphate (Melford Laboratories Ltd, Ipswich, UK) and 350μmol/L zeocin (Invitrogen). For the establishment of monoclonal lines, single colonies were picked after growth under selective pressure 3–4 weeks after transfection and propagated to establish monoclonal stable cell lines.

Animal Husbandry

All animal facilities and suppliers have been approved by the UK Home Office Licensing Authority and meet all current regulations and standards for the UK. Animals were bred in our on-site biological services unit and housed in groups of 4-6 per individually ventilated cage (IVC; Allentown Europe, UK), in a 12 h light dark cycle (06:30-18:30 light; 18:30-06:30 dark), with controlled room temperature (21 ± 1◦C) and relative humidity (40–60%). Animals remained in the same social group throughout the study with ad libitum access to standard diet and water. Mice were studied between 12-24 weeks of age with equal sex distribution. Blinding was performed during measurements and data processing.

Generation of Kir6x global knockout mice

The generation of Kir6.1 global knockout mice (6.1gKO) has been described in detail previously.[16] Briefly, in collaboration with Genoway (Lyon, France; project number genOway/EV/TIN1- *Kcnj8* 070206), we targeted exon 2 of the mouse *Kcnj8* gene. Using homologous recombination, a loxP site together with a FRT flanked neomycin selection cassette was inserted within intron 1 upstream of exon 2. Exon 2 contains the ATG translation initiation codon and another loxP was inserted distal to exon 2 in intron 2. The targeting construct was transfected into E14Tg2a embryonic stem (ES) cells derived from 129P2/Ola mice and one positive clone was isolated that had the intended 5' and 3' homologous recombination event in one allele. There were no other randomly integrated copies of the targeting vector. This ES cell clone was injected into C57Bl/6J blastocysts and implanted into pseudo-pregnant females. A number of male chimeras were bred with Flp deleter mice to remove the neomycin cassette and generate Kir6.1(flx/+) mice (Figure S7). The Kir6.1(flx/+) were crossed with C57Bl/Cre deleter mice (which ubiquitously express the cre recombinase) to develop mice with global genetic deletion of one allele of Kir6.1 (Kir6.1(+/-)). Kir6.1 (+/-)

were backcrossed onto a C57Bl/6 background for at least six generations. Homozygous Kir6.1 global KO (Kir6.1(-/-), 6.1-gKO) mice and littermate controls were generated by crossbreeding of the Kir6.1(+/-) heterozygous mice.

Global Kir6.2 KO mice (6.2-gKO) were produced with the help of The Medical Research Council Centre for Mouse Genetics, Harwell Campus, Oxfordshire, UK (MRC Harwell) in collaboration with the International Mouse Phenotyping Consortium (IMPC), the European Conditional Mouse Mutagenesis Program (EUCOMM) and the International Knockout Mouse Consortium (IKMC).[47] The process has been described previously by the IMPC.[47] Briefly, a construct containing a *lacZ* sequence followed by a neomycin-resistance cassette was integrated via homologous recombination into the *Kcnj11* gene upstream of its sole exon. The *lacZ* sequence together with the neomycin-resistance cassette were flanked by FRT sites and the neomycin-resistance cassette together with the *Kcnj11* exon flanked by loxP sites producing a construct termed tm1a (Figure S8). The construct was transfected into embryonic stem cells (ESCs) harvested from a developing blastocyst creating the ES cell clone (clone id EPD0974_3_G05). ESCs exhibiting the transfected sequence were selectively cultured in antibiotic containing media. The cultured ESCs were then injected back into a blastocyst and implanted into a pseudo-pregnant female. Chimeric tm1a pups were born and crossed with C57Bl/6 wild-type mice to enable germ line transmission. Thereafter tm1a sperm were isolated and in-vitro fertilisation (IVF) performed in the presence of soluble Cre thus mediating excision of the loxP flanked neomycin-resistance cassette and *Kcnj11* exon and generating pups heterozygous for the knockout tm1b allele (Kir6.1 (+/-)). When crossed these mice gave rise to litters containing pups with homozygous global knockout of Kir6.2 (Kir6.2(-/-), 6.2-gKO) and wild-type litter`mate controls. Mice were bred to order generating stock totalling wild-type 61 and gKO 58. Mice were housed at MRC facilities until 6 weeks of age

before being shipped and then acclimatised at our facilities until experimental age of 3-6 months.

Genotyping

DNA was extracted from mouse ear samples by proteinase K digestion. PCR to confirm Kir6.1 gene knock-out used the BioMix™ Red stable *Taq* DNA polymerase system (Bioline Reagents Ltd, UK) and the following primers: For the wild-type allele - sense 5' ACTAGCACCTCTATCCCCAGCTCCTACC 3' and antisense 5' CCGCCCCTCCCTCTGAACCTATATC 3' and for the knock-out allele – sense 5' ACTAGCACCTCTATCCCCAGCTCCTACC 3' and antisense 5' CTGACACCAAAGCTGCCTGACAACA 3'. PCR cycle conditions were denaturation at 94°C for 2 minutes, 35 cycles of 94°C for 30 secs, 65°C for 30 secs, 68°C for 40 secs, extension at 68°C for 8 minutes. This yielded a WT allele band of 474 bp and a knock-out allele band of 724 bp. Failure of knock-out leads to a product between the two primers too large to PCR under standard conditions (Figure S9).

Confirmation of Kir6.2 gene knockout was performed by The Medical Research Council Centre for Mouse Genetics, Harwell Campus, Oxfordshire, UK (MRC Harwell) in collaboration with the International Mouse Phenotyping Consortium (IMPC) utilising qPCR of gDNA.[47] All qPCR assays were FAM labelled using GTX Taqman master mix (Applied Biosystems) and run in duplex with a VIC labelled internal control, Dot1l (Primer 1 = GCCCCAGCACGACCATT, Primer 2 = TAGTTGGCATCCTTATGCTTCATC, Probe = CCAGCTCTCAAGTCG). Signal is calibrated against controls with known allele copy number. A LacZ assay designed around the sequence of the LacZ reporter (Primer 1 = CTCGCCACTTCAACATCAAC, Primer 2 = TTATCAGCCGGAAAACCTACC, Probe = TCGCCATTTGACCACTACCATCAATCC) is used in conjunction with a neomycin assay

designed around the sequence of the neomycin resistance cassette (Primer 1 = GGTGGAGAGGCTATTCGGC, Primer 2 = GAACACGGCGGCATCAG, Probe = TGGGCACAACAGACAATCGGCTG) and 2 WT assays designed around the WT breakpoint sequence that is lost around the loxP sites of the synthetic cassette (Primer 1 = GGGCACGTGGAAAGTGAAG, Primer 2 = AGCCGAGCAGGGTCTTGTC, Probe = TAGAGTGGTGGGGTGCGAGC), and an assay designed around the critical sequence flanked by the two loxP sites (Primer 1 = ACGACCTGGCTCCTAGTGA, Primer2 = ACCACGCCTTCCAAGATGAC, Probe = CTGCACCACCACCAGGACCTG) (Figure S8). The presence of a tm1b knockout allele is driven only by a positive copy number from the LacZ assay and no copy number from any other assay. The presence of the WT allele is driven only by a positive copy number from the WT breakpoint sequence and WT critical sequence assays and no copy number from the other assays.

Quantitative RT-PCR

Total RNA was extracted from mouse tissues using the RNAeasy kit from Qiagen.

Commercially available isolated human total RNA from all four heart chambers (right atrium, left atrium, right ventricle and left ventricle) from three separate donors and said to be normal with no past medical history and normal cardiac structure and function, was purchased from AMS Biotechnology (Europe) Limited. Donors were a 49 year old male, 69 year old male and 65 year old male.

Total RNA was DNase I-treated and reverse-transcribed using the high-capacity cDNA reverse transcription kit (Applied Biosystems).

Quantitative RT-PCR was performed with 90 ng of murine cDNA and 50 ng of human cDNA using customized TaqMan gene expression assays (Applied Biosystems). We used the commercially available probes for all the genes as listed below: Mouse - Mm00434620_m1 for

Kcnj8 (Kir6.1), Mm00440050_s1 for *Kcnj11* (Kir6.2), Mm00803450_m1 for *Abcc8* (SUR1) and Mm00441638_m1 for *Abcc9* (SUR2), Mm00724407_m1 for *AnoI*, Mm01188822_m1 for *Cacna1c*, Mm00486572_m1 for *Cacna1g*, Mm00445382_m1 for *Cacna1h*, Mm00434584_s1 for *Kcna2*, Mm00492791_m1 for *Kcnb1*, Mm03057813_m1 for *Kcnb2*, Mm00434616_m1 for *Kcnj2*, Mm00440058_s1 for *Kcnj12*, Mm00516078_m1 for *Kcnma1*, Mm01342518_m1 for *Scn5a*, Mm01255747_g1 for *Nppa*, Mm01255770_g1 for *Nppb*, Mm00600555_m1 for *Myh7*, Mm01192933_g1 for *Ctgf*, Mm01178820_m1 for *Tgfb1*, Mm00436955_m1 for *Tgfb2*, Mm00801666_g1 for *Col1a1*, Mm00802305_g1 for *Col3a1*. Human - Hs00958961_m1 for *KCNJ8* (Kir6.1), Hs00265026_s1 for *KCNJ11* (Kir6.2), Hs01093770_m1 for *ABCC8* (SUR1), Hs02514422_s1 for *ABCC9* transcript variant SUR2A, Hs02515384_s1 for *ABCC9* transcript variant SUR2B. Each gene was assayed in triplicate. Relative expression was calculated by using the comparative C_T method normalized to GAPDH. The final data are presented as a relative change compared with control.

Histology and immunohistochemistry

Mouse hearts collected from 10-12 week old animals were rinsed thoroughly in PBS to remove excess blood and fixed in 10 % formalin for at least 24 hours. After the fixation process, they were washed twice in PBS and stored in 70 % ethanol before paraffin embedding. Paraffin-embedded myocardium were cut at 5 μm thick sections and mounted on clear, plus microscope slides. For histological analysis, sections were stained for haematoxylin and eosin with automated Leica autostainer XL system (*Leica Biosystems, UK*) and Trichrome stain kit (*Ab150686, Abcam, UK*) to detect cardiac fibrosis.

Kir6.1 and Kir6.2 global samples from the various histological stains were scanned with the Pannoramic 250 High Throughput Scanner (*HistoTech, Budapest, Hungary*) and representative images from WT and KO hearts with scale bars were processed with Panoramic

Viewer software (*HistoTech, Budapest, Hungary*).

Isolation of murine atrial myocytes

Mice were killed via cervical dislocation, the hearts were rapidly excised, and the left and right atria dissected and placed in Tyrode's solution consisting of 140 mmol/L NaCl, 5.4 mmol/L KCl, 1.8 mmol/L $CaCl_2$, 1 mmol/L $MgCl_2$, 5 mmol/L HEPES-NaOH, and 5.5 mmol/L D-glucose (adjusted to pH 7.4 with NaOH). Piece-meal atria were then placed in low Mg^{2+} / Ca^{2+} solution containing 140 mmol/L NaCl, 5.4 mmol/L KCl, 0.5 mmol/L $MgCl_2$, 0.2 mmol/L $CaCl_2$, 1.2 mmol/L KH_2PO_4, 50 mmol/L taurine, 5.5 mmol/L D-glucose, 1.0 mg/ml BSA, and 5.0 mmol/L HEPES-NaOH (adjusted to pH 6.9 with NaOH). The tissue was digested with 1 mg/ml collagenase type II (Worthington), 1 mg/ml protease (Sigma Aldrich), 1 mg/ml BSA (Sigma Aldrich), and 400 µmol/L $CaCl_2$ for ~20 min at 37 °C with consistent agitation with a fire-polished Pasteur pipette. The tissue was then washed three times in modified Kraft–Brühe solution containing 70 mmol/L L-glutamic acid, 20 mmol/L KCl, 80 mmol/L KOH, 10 mmol/L KH_2PO_4, 10 mmol/L taurine, 1 mg/ml BSA, and 10 mmol/L HEPES-KOH, pH 7.4 with KOH. Atrial cells were dissociated manually using a fire-polished Pasteur pipette in Kraft–Brühe solution and allowed to rest for 5 min prior to re-adaptation. The cells were allowed to re-adapt to physiological levels of Na^+ and Ca^{2+} by incremental addition of a solution containing 10 mmol/L NaCl and 1.8 mmol/L $CaCl_2$ and finally normal Tyrode's solution with 1 mg/ml BSA. Cells were then plated on laminin-coated 10 mm coverslips and left for 30 minutes at 37°C in Tyrode's solution with 1 mg/ml BSA prior to patch clamp recordings.

Patch clamp electrophysiology

Whole-cell patch-clamp recordings were performed as described previously.[48] Pipette resistance varied between 2-3MΩ. Capacitance transients and series resistance in whole-cell

recordings were compensated electronically by using amplifier circuitry (Axopatch 200B, Molecular Devices). The data were filtered at 2 kHz (4-pole Bessel) and sampled at 10 kHz using a Digidata 1440 (Molecular Devices). The currents were acquired and analysed using pClamp10 (Molecular Devices). Whole-cell currents were recorded using a voltage-ramp protocol (-150 to +50 mV over 1 s at 0.1 Hz). Pipette solution contained 110 mmol/L KCl, 1 mmol/L $MgCl_2$, 10 mmol/L EGTA, 10 mmol/L HEPES, 3 mmol/L MgATP and 1 mmol/L Na_2ADP, pH 7.2 using KOH. The bath solution contained 130 mmol/L NaCl, 4 mmol/L KCl, 1 mmol/L $MgCl_2$, 1.8 mmol/L $CaCl_2$, 10 mmol/L D-glucose, 10 mmol/L HEPES, pH 7.35 using NaOH. Whole-cell action potentials were recorded using the current-clamp configuration. Action potentials were triggered by 600 pA/5ms square pulses at 1 Hz. The pipette solution for current-clamp recordings contained 110 mmol/L potassium gluconate, 20 mmol/L KCl, 10 mmol/L HEPES, 0.05 mmol/L EGTA, 0.5 mmol/L $MgCl_2$, 5 mmol/L MgATP, 0.3 mmol/L Na_2-GTP, 5 mmol/L Na_2-phosphocreatine, pH 7.4 using KOH. The data were initially analysed using Clampfit 10 (Axon Instruments). Drugs were applied to the bath using a gravity-driven perfusion system having been dissolved in bath solution fresh from stocks (diazoxide, pinacidil, tolbutamide, glibenclamide, HMR1098 in ≥99.5% dimethylsulfoxide; PNU37883 in H_20) on the day of each experiment.

Supplemental Results

K_{ATP} pharmacology

HEK293 stable lines expressing K_{ATP} channels of all homomultimeric Kir6x/SURx combinations demonstrated selective pharmacology to whole-cell patch clamp. Diazoxide 100μmol/L (DZX) strongly activated SUR1-containing channels (Figure S1A & S1E to H:

Kir6.1: $p<0.001$, $n=5$; Kir6.2: $p<0.0001$, $n=5$) but failed to activate those containing SUR2A (Figure S1B). For pinacidil 10μmol/L (PIN) the opposite was true in that it failed to activate SUR1-containing channels (Figure S1A, S1E & S1F) but strongly activated SUR2A-containing channels (Figure S1B: Kir6.1: $p<0.05$, $n=6$; Kir6.2: $p<0.0001$, $n=5$). DZX activated SUR2B-containing channels (Figure S1C: Kir6.1: $p<0.05$, $n=5$; Kir6.2: $p<0.001$, $n=5$) but PIN did so more strongly (Figure S1C: PIN vs DZX: Kir6.1: $p<0.05$, $n=5$; Kir6.2: $p<0.0001$, $n=5$).

We have demonstrated that PNU selectively blocks Kir6.1 over Kir6.2. When SUR1-containing channels were DZX-activated and SUR2A- and SUR2B-containing channels PIN-activated, PNU37883 50μmol/L (PNU) selectively inhibited Kir6.1-containing channels (Figure S1D: ~90% current inhibition of that produced with TOLB for SUR1-containing channels and glibenclamide 10μmol/L (GLIB) for SUR2A- and SUR2B-containing channels). PNU did not inhibit Kir6.2-containing channels, (Figure S1D: ~0-20% inhibition; Kir6.1 vs Kir6.2: $p<0.0001$, $n=5$ for all homomultimeric compositions).

Neither DZX or PIN activated current in untransfected HEK293 cells (Figure S1I to K). PNU was able to inhibit a small baseline current of ~15pA/pF in untransfected HEK293 cells (Figure S1I to K: $p<0.05$ vs baseline, $n=5$). Glibenclamide is a non-selective K_{ATP} channel inhibitor acting at the SUR subunit.[3] Demonstration of the inhibition afforded by glibenclamide 10μmol/L (GLIB) in HEK293 stable cell lines is shown in Figure S1B & S1C. However, glibenclamide 10μmol/L (GLIB) failed to inhibit a current in untransfected HEK293 cells like PNU (Figure S1I to S1K). This would suggest that PNU has off-target effects in addition to its inhibitory effects on Kir6.1-containing channels.

There are conflicting data as to whether HMR1098 is a selective inhibitor of SUR2A-containing channels.[49] We have shown it strongly inhibited, ~100% inhibition, Kir6.2/SUR1 and Kir6.2/SUR2A channels and could not distinguish between SUR1 and SUR2A (Figure S2A to D). Tolbutamide 100μmol/L (TOLB) did not strongly inhibit Kir6.2/SUR2A channels,

~25% inhibition (Figure S2B & D). TOLB selectively inhibited SUR1-containing channels after preceding activation by DZX, regardless of the pore forming subunit in complex, (Figure S1A: Kir6.1: $p<0.0001$, $n=5$; Kir6.2: $p<0.0001$, $n=5$).

Supplemental Tables

Supplemental Table I

**Parameter**	**WT**	**6.1-gKO**	**6.2-gKO**
Baseline SN firing rate (bpm)	391±21 *(20)*	329±20 *(15)* *	429±41 *(8)*
Baseline ERP (ms)	26±2 (*20)*	47±5 *(15)* [‡]	42±4 *(8)* [†]
8mins hypoxia ERP (ms)	21±2 *(16)*	46±9 *(12)* *	74±11 *(8)* [‡ §]
12mins hypoxia ERP change from baseline (%)	-9±9 *(11)*	-41±11 *(4)*	+114±46 *(7)* [‖]
12mins hypoxia + TOLB ERP change from baseline (%)	+74±33 *(6)* [#]	+56±20 *(7)* **	-

Sinus node (SN) firing rate and effective refractory period (ERP) measurements from Langendorff mouse hearts. The data are shown as mean±SEM. *(n)* hearts. *$p<0.05$ vs WT, [†]$p<0.001$ vs WT, [‡]$p<0.0001$ vs WT, [§]$p<0.05$ vs 6.1-gKO by one-way ANOVA with Tukey's post-test. [‖]$p<0.0001$ vs WT by two-way ANOVA with Dunnett's post-test. [#]$p<0.0001$ vs WT no TOLB, **$p<0.01$ vs 6.1-gKO no TOLB by two-way ANOVA with Bonferroni's post-test.

Supplemental Table II

Parameter	WT	6.1-gKO	6.2-gKO
Baseline steady state CV (m/s)	0.66±0.04 *(19)*	0.52±0.06 *(13)*	0.56±0.03 *(8)*
Baseline S2-60ms CV (m/s)	0.52±0.04 *(19)*	0.32±0.06 *(8)* *	0.31±0.06 *(7)* *
8mins hypoxia steady state CV (m/s)	0.46±0.04 *(15)*	0.32±0.05 *(10)* *	0.30±0.04 *(8)* *
8mins hypoxia S2-60ms CV (m/s)	0.41±0.04 *(15)*	0.19±0.03 *(7)* †	0.17±0.06 *(3)* *
12mins hypoxia S2-60ms CV (m/s)	0.30±0.05 *(10)*	-	-
12mins hypoxia + TOLB S2-60ms CV (m/s)	0.27±0.02 *(5)*	-	-
12mins hypoxia CV change from baseline (%)	-47±6 *(10)* ‡	-40±15 *(4)* §	-53±7 *(7)* ‡
12mins hypoxia + TOLB CV change from baseline (%)	-39±4 *(6)*	-33±11 *(5)*	-

Conduction velocity (CV) measurements from Langendorff mouse hearts. The data are shown as mean±SEM. *(n)* hearts. *p<0.05 vs WT, †p<0.01 vs WT, ‡p<0.0001 vs baseline, §p<0.05 vs baseline by two-way ANOVA with Dunnett's post-test.

Supplemental Table III

**Parameter**	**WT**	**6.1-gKO**	**6.2-gKO**
Baseline WFPL (cm)	1.7±0.2 *(19)*	2.5±0.4 *(13)* *	2.3±0.3 *(8)* *
8mins hypoxia WFPL (cm)	0.9±0.1 *(15)*	1.6±0.5 *(10)*	2.3±0.5 *(8)* †
12mins hypoxia WFPL change from baseline (%)	-49±8 *(10)* ‡	-66±11 *(4)* §	4±27 *(7)* ‖
12mins hypoxia + TOLB WFPL change from baseline (%)	+1±13 *(6)* #	+8±22 *(5)* **	-

Wavefront path length (WFPL) measurements from Langendorff mouse hearts. The data are shown as mean±SEM. *(n)* hearts. *$p<0.05$ vs WT, †$p<0.001$ vs WT by one-way ANOVA with Tukey's post-test. ‡$p<0.001$ vs baseline, §$p<0.01$ vs baseline, ‖$p<0.01$ vs WT by two-way ANOVA with Dunnett's post-test. #$p<0.05$ vs WT no TOLB, **$p<0.05$ vs 6.1-gKO no TOLB by two-way ANOVA with Bonferroni's post-test.

Supplemental Figures

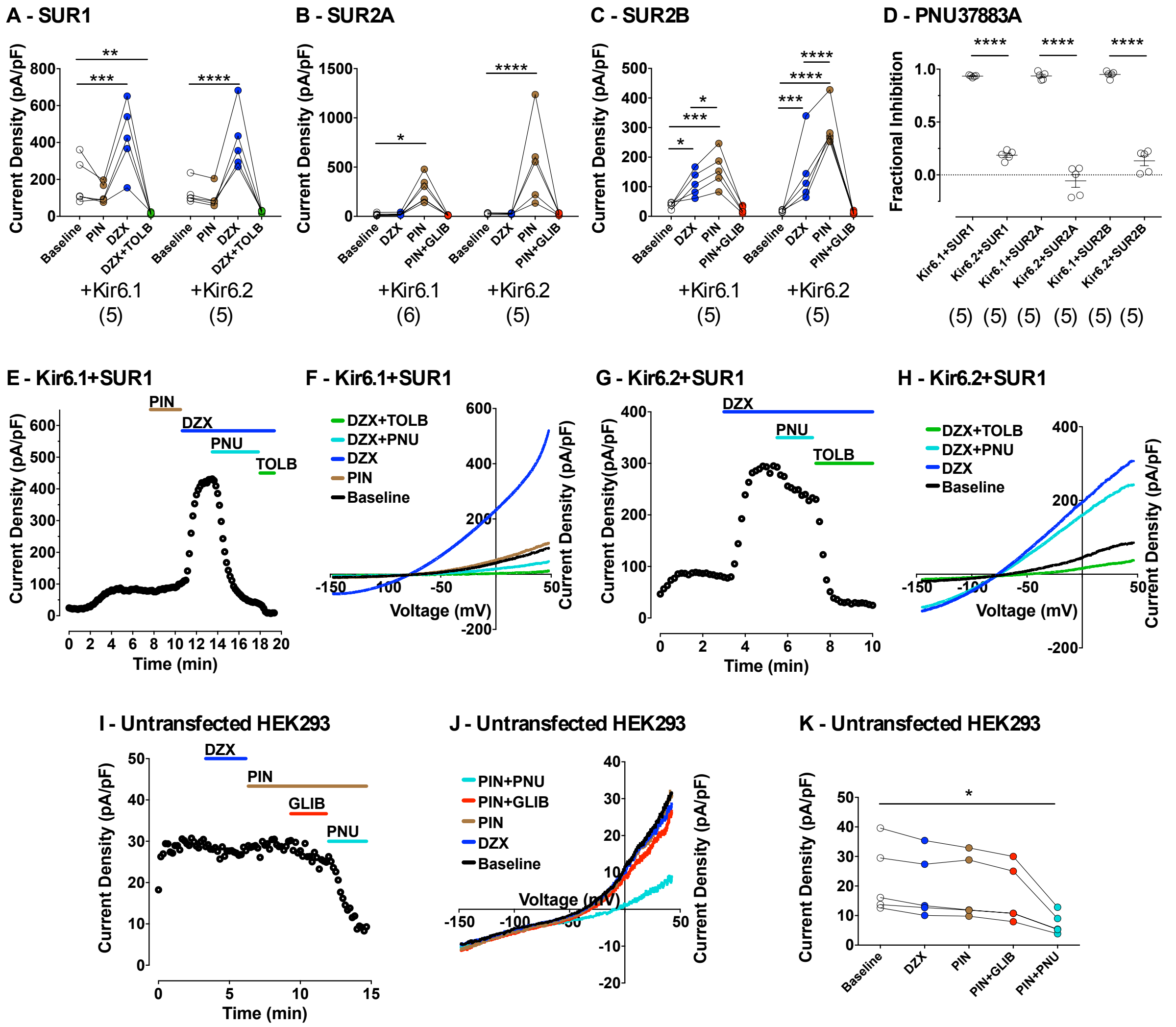

Supplemental Figure I. K_{ATP} pharmacological fingerprint in HEK293 stable cell lines. (A to C) Repeated measures whole-cell current densities at +40 mV and differential response to K_{ATP} openers pinacidil 10 µmol/L (PIN, brown circles), diazoxide 100 µmol/L (DZX, blue circles) and inhibitors tolbutamide 100 µmol/L (TOLB, green circles), glibencamide 10 µmol/L (GLIB, red circles) for cells transfected with Kir6x/SUR1 channels (A), Kir6x/SUR2A channels (B), Kir6x/SUR2B channels (C). (*n*) cells. *P* vs baseline unless indicated by repeated measures two-way ANOVA with Dunnett's post-test. (D) Differential inhibition by

PNU37883A 50μM (PNU) of K_{ATP} current (mean±SEM, *n*=5 each cell line, *P* by one-way ANOVA with Bonferroni's post-test). (E) Representative time-course trace at +40 mV and (F) whole-cell current density-voltage traces for cell expressing Kir6.1/SUR1. (G) Representative time-course traces at +40 mV and (H) whole-cell current density-voltage traces for cell expressing Kir6.2/SUR1 channels. (I) Representative time-course trace at +40 mV and (J) whole-cell current density-voltage traces of an untransfected HEK293 cell. (K) Repeated measures data of whole-cell current densities at +40 mV for untransfected HEK293 cells. (*n*=5 cells). *$p<0.05$, **$p<0.01$, ***$p<0.001$, ****$p<0.0001$.

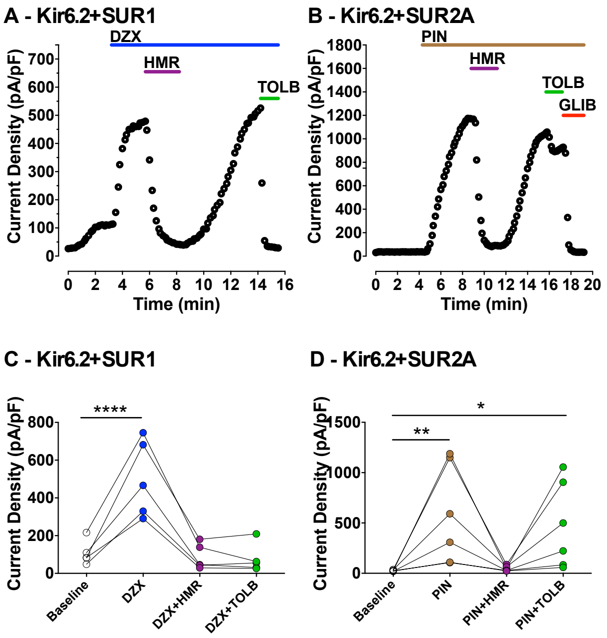

Supplemental Figure II. Tolbutamide specificity for SUR1 containing channels. Representative time-course traces at +40 mV are shown of (A) cell expressing Kir6.2/SUR1 channels and (B) cell expressing Kir6.2/SUR2A channels. (C) Repeated measures whole-cell current densities at +40 mV and differential response to the K_{ATP} opener diazoxide 100 µmol/L (DZX, blue circles) and inhibitors HMR1098 10µmol/L (HMR, purple circles) and tolbutamide 100 µmol/L (TOLB, green circles) for cells expressing Kir6.2/SUR1 channels. (*n*=5 cells). (D) Repeated measures whole-cell current densities at +40 mV and differential response to the K_{ATP} opener pinacidil 10µmol/L (PIN, brown circles) and inhibitors HMR1098 10µmol/L (HMR, purple circles) and tolbutamide 100 µmol/L (TOLB, green circles) for cells expressing Kir6.2/SUR2A channels. (*n*=6 cells). *P* vs baseline by repeated measures two-way ANOVA with Dunnett's post-test. *p<0.05, **p<0.01, ***p<0.001, ****p<0.0001.

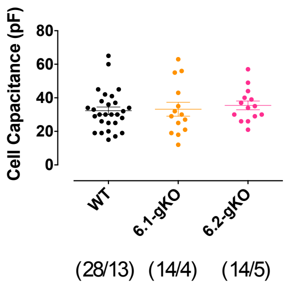

Supplemental Figure III. Cell capacitance of isolated murine atrial myocytes. Cell capacitance as a surrogate of cell size. Data are shown as mean±SEM and compared using one-way ANOVA with Tukey's post-test. (*n* cells/*n* mice).

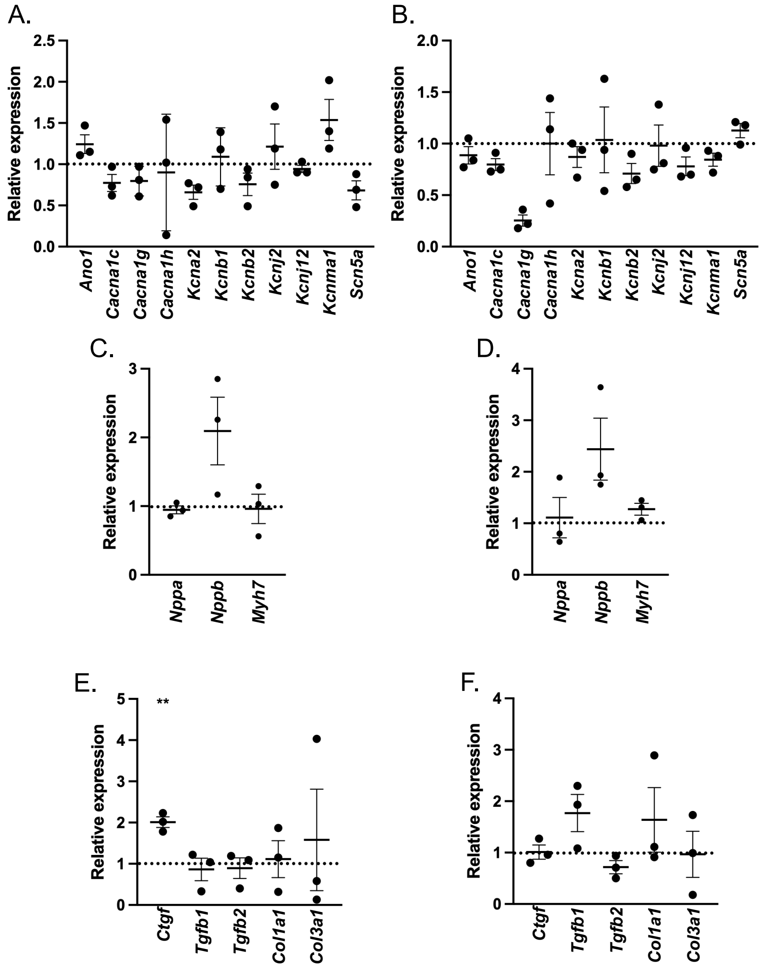

Supplemental Figure IV. Comparison of cardiac ion channel and structural marker expression in Kir6.1 global knockout hearts. Normalised qPCR results of 6.1-gKO atria using ion channel genes (A), hypertrophy markers (C) and fibrosis markers (E) and global KO ventricle using ion channel genes (B), hypertrophy markers (D) and fibrosis markers (F). KO values (n=3) are calculated by normalising to a WT (n=3) value of 1, represented by the dotted line. The mean±SEM is indicated. Students t-test was used to calculate significance compared to WT. ** p<0.01.

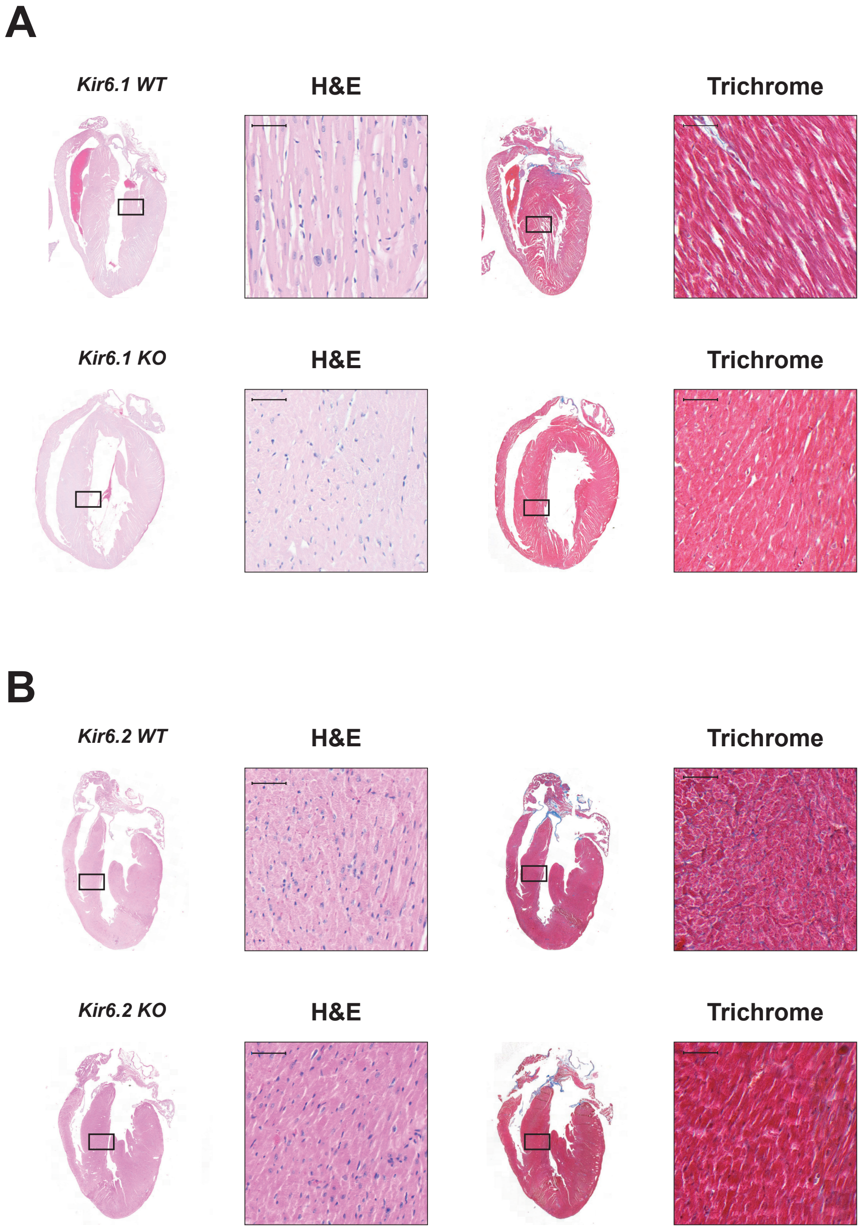

Supplemental Figure V. Histological analysis of Kir6.1 and Kir6.2 global knockout mouse hearts. Examples of WT and KO hearts from each transgenic mouse line are shown. (A) H&E staining shows no abnormal morphology in Kir6.1 WT and KO hearts, all 4 chambers appear normal. Masson's Trichrome stain was also used to detect collagen deposition with no differences between WT and KO. (B) No morphological or structural differences were observed in the Kir6.2 WT and KO hearts. These results confirm there are no signs of

replacement fibrosis or myocyte loss for both transgenic murine models. (Scale bar denotes 50 μm, black squares denote the magnified region).

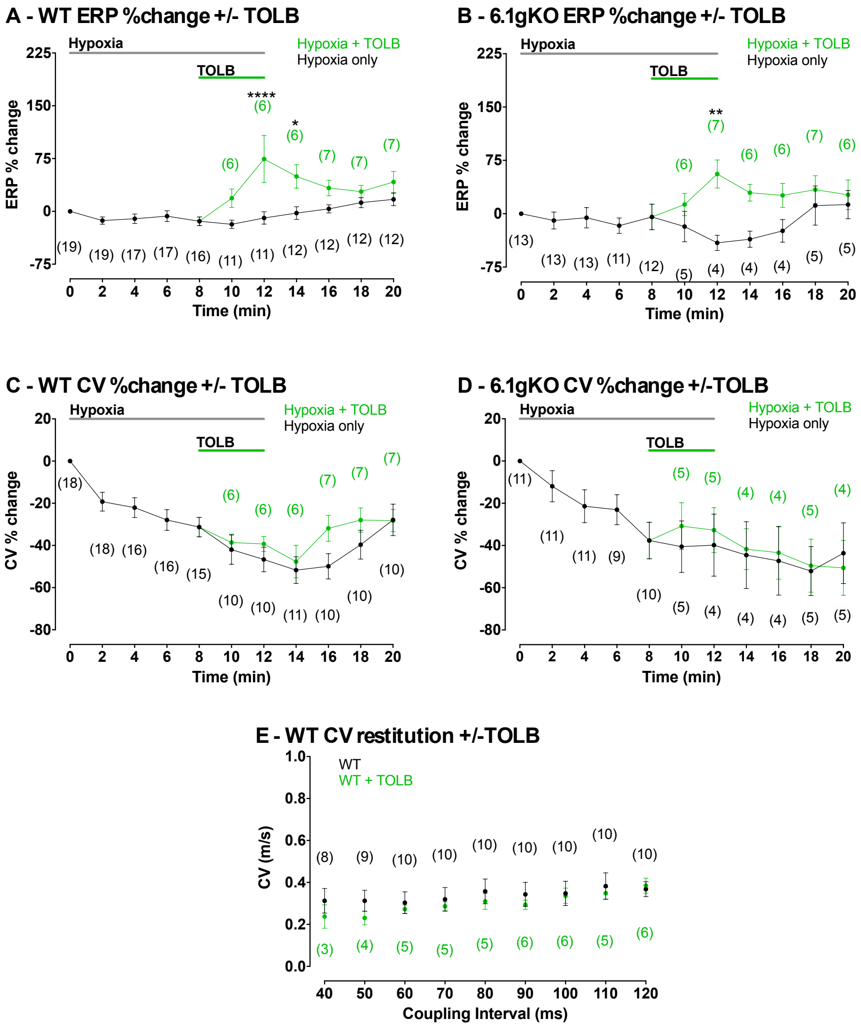

Supplemental Figure VI. Effects on atrial effective refractory period (ERP) and conduction velocity (CV) of K_{ATP} pharmacological inhibition in Langendorff mouse hearts. (A) ERP change normalised to baseline during hypoxia in WT mice with and without K_{ATP} inhibition. (B) As for (A) but for 6.1-gKO mice. (C) CV change normalised to baseline during hypoxia in WT mice with and without K_{ATP} inhibition. (D) as for (C) but for 6.1 gKO mice. (E) WT mice CV restitution at 12 minutes of hypoxia with and without K_{ATP} inhibition. Data are shown as mean±SEM, (*n*), by two-way ANOVA with Bonferroni's post-test. (TOLB, tolbutamide 100 μmol/L). **p*<0.05, ***p*<0.01, *****p*<0.0001.

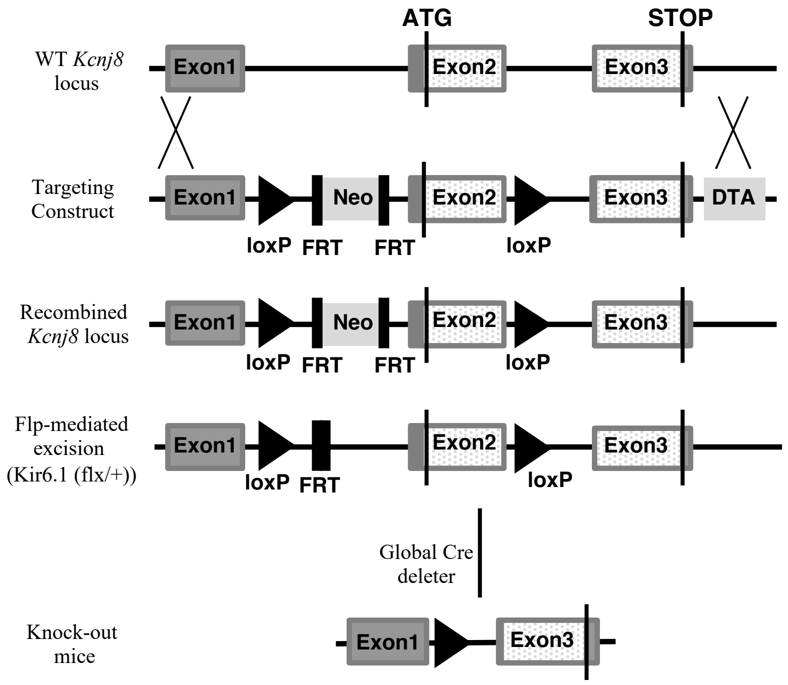

Supplemental Figure VII. Targeting strategy using Cre-loxP technology for the deletion of exon 2 of the *Kcnj8* gene. The targeting vector construct for *Kcnj8* included a 5' loxP site and a FRT-flanked neomycin (Neo) selection cassette within intron 1 upstream of exon 2 and the second loxP site in intron 2 with a diptheria toxin A (DTA) negative selection marker downstream. Transfected embryonic stem (ES) cells homologously recombined the inserted targeting construct and were injected into a developing blastocyst. Mice with the engineered *Kcnj8* locus were crossed with global flp-deleter mice to allow Flp-mediated excision of the neomycin selection cassette and generate Kir6.1 (flx/+) offspring. The Kir6.1(flx/+) were crossed with C57Bl/Cre deleter mice to develop mice with global genetic deletion of one allele of Kir6.1 (Kir6.1(+/-)). Kir6.1 (+/-) were backcrossed onto a C57Bl/6 background for at least six generations. Homozygous Kir6.1 global KO (Kir6.1(-/-), 6.1-gKO) mice and littermate controls were generated by crossbreeding of the Kir6.1(+/-) heterozygous mice.

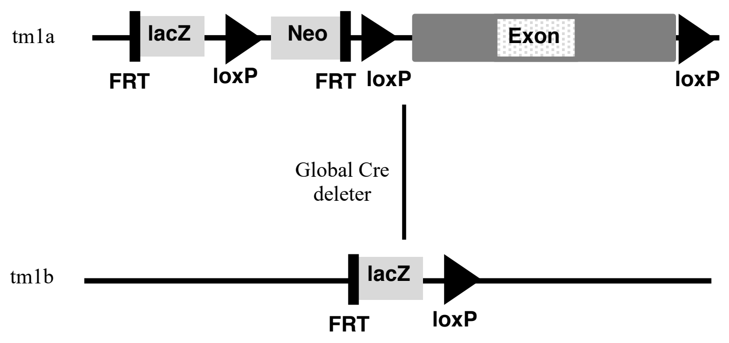

Supplemental Figure VIII. Targeting strategy using Cre-loxP technology for the deletion of the exon of the *Kcnj11* gene. A construct containing a *lacZ* sequence followed by a neomycin-resistance cassette was integrated via homologous recombination into the *Kcnj11* gene upstream of its sole exon. The *lacZ* sequence together with the neomycin-resistance cassette were flanked by FRT sites and the neomycin-resistance cassette together with the *Kcnj11* exon flanked by loxP sites producing a construct termed tm1a. ES cells transfected with this construct were injected into a developing blastocyst and tm1a sperm isolated and IVF performed in the presence of soluble Cre producing mice heterozygous for the KO tm1b allele. When crossed these mice gave rise to Kir6.2 global KO (Kir6.1(-/-), 6.2-gKO) mice.

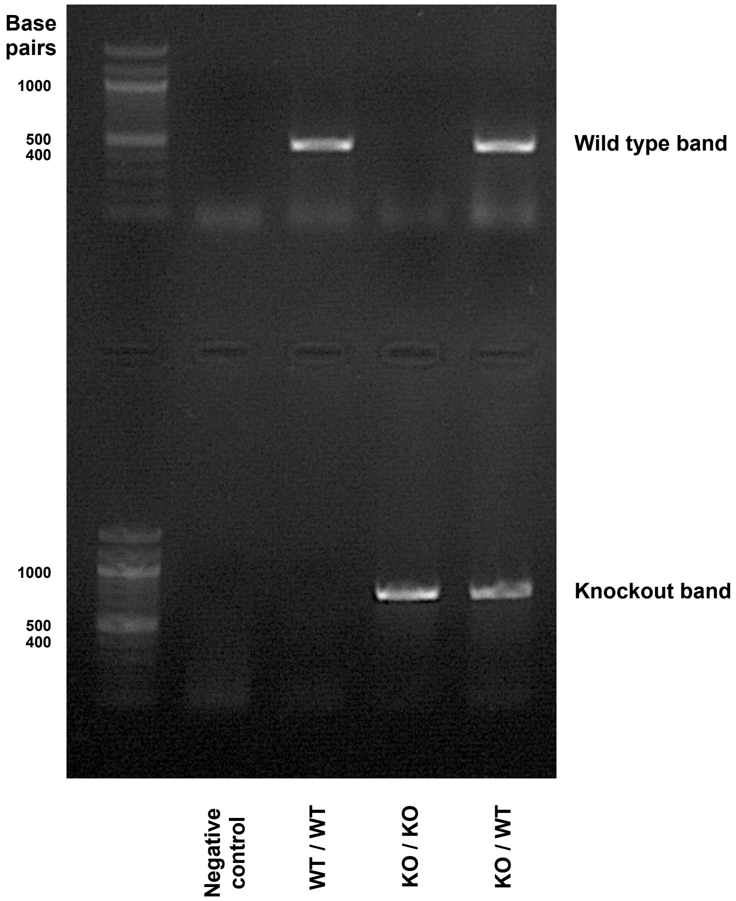

Supplemental Figure IX. Genomic DNA PCR for the Kir6.1 global knock-out mouse. Top half shows WT band (474bp product) and bottom half KO band (724bp) product. Failure of knock-out leads to a product between the two knock-out assay primers too large to PCR under standard conditions.
